# Modelling drought in South Africa: meteorological insights and predictive parameters

**DOI:** 10.1007/s10661-024-13009-y

**Published:** 2024-09-21

**Authors:** Nnaemeka Onyeuwaoma, Venkataraman Sivakumar, Mahesh Bade

**Affiliations:** 1 Discipline of Physics, School of Chemistry and Physics, College of Agriculture, Engineering and Science, University of KwaZulu Natal, Westville Campus, Durban, 4001 South Africa; 2 National Institute for Theoretical and Computational Sciences, University of KwaZulu Natal, Westville Campus, Durban, 4001 South Africa

**Keywords:** Drought, Simulations, Precipitation, South Africa, SPI

## Abstract

South Africa has grappled with recurring drought scenarios for over two decades, leading to substantial economic losses. Droughts in the Western Cape between 2015 and 2018, especially in Cape Town was declared a national disaster, resulting in the strict water rationing and the “day zero” effect. This study presents a set of simulations for predicting drought over South Africa using Artificial Neural Network (ANN), using Standard Precipitation Index (SPI) as the drought indicator in line with the recommendations of the World Meteorological Organization (WMO). Furthermore, different meteorological variables and an aerosol parameter were used to develop the drought set in four distinct locations in South Africa for a 21-year period. That data used include relative humidity (rh), temperature (tp), soil wetness (sw), evapotranspiration (et), evaporation (ev) sea surface temperature (st), and aerosol optical depth (aa). The obtained *R*^2^ values for SPI3 ranged from 0.49 to 0.84 and from 0.22 to 0.84 for SPI6 at Spring Bok, Umtata 0.83 to 0.95 for SPI3, and 0.61 to 0.87 for SPI6; Cape Town displayed *R*^2^ values from 0.78 to 0.94 for SPI3 and 0.57 to 0.95 for SPI6, while Upington had 0.77–0.95 for SPI3, and 0.78–0.92 for SPI6. These findings underscore the significance of evapotranspiration (et) as a pivotal parameter in drought simulation. Additionally, the predictive accuracy of these parameter combinations varied distinctly across different locations, even for the same set of parameters. This implies that there is no single universal scheme for drought prediction. Hence, the results are important for simulating future drought scenarios at different parts of South Africa. Finally, this study shows that ANN is an effective tool that can be utilized for drought studies and simulations.

## Introduction

Drought, recognized as a prolonged and extensive scarcity of natural water resources, is a terrible natural phenomenon with far-reaching consequences (WHO, 2023; USGS, 2023). Its occurrence is not confined to specific regions, impacting diverse areas across the globe (Hao, et al., 2018). However, the manifestation and severity of drought exhibit distinct characteristics contingent upon regional climates, resulting in varying impacts on societies and environments (WMO, 2012). This period is majorly characterized with dry weather because of low rainfall and high temperatures. According to Heim (2002), the consequences of drought encompass multifaceted domains such as health, agriculture, economies energy, and the environment. Hence, the interplay between drought and climate change is increasingly evident, with rising temperatures exacerbating the severity and frequency of drought occurrences (Mukherjee, et al., 2018; Shamshirband, et al., 2020). Also, variations in climate pattern such as El Niño and La Niña can lead to drought. El Nino which is characterized by warmer ocean temperatures can increase the occurrence of drought in the USA and Southern Africa, while La Nina associated with cooler oceanic temperatures can lead to drought in Australia (Rescue, 2023). Furthermore, changes in the jet stream can also cause drought when dry air from one part of the globe is brought to other areas.

Furthermore, anthropogenic activities, including urbanization, deforestation, and alterations in land use, have compounded drought effects (Deo, et al., 2017; IPCC, 2012; McAlpine, et al., 2009). These changes modify the energy balance within ecosystems, leading to increased aridity, evapotranspiration, soil albedo, and temperatures. Despite concerted efforts, drought remains one of the most complex and least understood natural hazards with far-reaching consequences (see Obasi, 1994; Kiem, et al., 2016; Sundararajan, et al., 2021). Studies have shown that the consequences of drought encompass negative effects on the environment, health, agriculture, energy, and the economy. These impacts stem from the pivotal role water plays in the everyday existence of all living organisms, including humans. This requires effective mitigation strategies against the impacts, while necessitating comprehensive approaches involving water conservation, improved agricultural practices, and the development of drought-resistant crops.

Consequently, implementing early warning systems equipped with models and drought contingency plans, often referred to as “drought response protocols,” is imperative across all sectors of the economy. This underscores the need for constant drought prediction and modelling which are crucial aspects of effective awareness, monitoring, and mitigation strategy. Addressing these aspects can significantly reduce the detrimental impacts of drought on society, both in the short and long term. Effective drought monitoring necessitates the utilization of robust modelling techniques, instrumental in devising mitigation strategies (Gorgij, et al., 2022). This involves establishing correlations between the indices of interest and predictor variables (Hao, et al., 2018). However, the intricate and nonlinear nature of drought poses significant challenges to modelling this phenomenon coupled with the fact that there is not a universally superior model applicable to all locations. Hence, the dynamic nature of drought modelling variables, influenced by environmental disparities, presents a substantial hurdle. As a result, most models are localized, relying on historical or re-analysis data to derive predictors (Hao et al., 2018). A range of models has been developed globally to forecast drought, each exhibiting varying degrees of accuracy, in line with the techniques used and geographical location. For instance, Shamshirband et al. (2020) predicted the SPI stream run-off using Gene Expression Programming (GEP), M5 model tree (M5), and Support Vector Regression (SVR), obtaining different *R*^2^ values: 0.396 and 0.691 for GEP, 0.701 and 0.829 for M5, and 0.853 and 0.898 for SVR. Rafiei-Sardooi et al. (2018) modelled drought in Iran employing ARIMA and neuro-fuzzy techniques, resulting in *R*^2^ values of 0.20 and 0.63 for SPI 3, and 0.50 and 0.79 for SPI 12, respectively. Adnan et al. (2021) utilized a hybrid Random Vector Functional Link-Hunger Games Search (RFVL–HGS) approach, yielding an accuracy of *R*^2^ = 0.815 for SPI 3 and *R*^2^ = 0.829 for SPI 6. Similarly, Gorgij et al. (2022) in Iran reported correlations ranging from 0.836 to 0.856 for SPI 3 and 0.889 to 0.909 for SPI 6 using Long Short-term Memory (LSTM). Khan et al. (2020) employed SVM, ANN, and KNN to categorize drought in Pakistan, obtaining varied correlation ranges for different drought classifications. Furthermore, Mouatadida et al. (2018) compared models and found that ANN outperformed extreme learning machine (ELM), multiple linear regression (MLR), and support vector regression (LSSVR) in predicting drought in Eastern Australia. Deo et al. (2017) combined various parameters and models achieving *R*^2^ values ranging from 0.994 to 0.989, 0.944 to 0.988, and 0.916 to 0.987 using MARS, LSSVM, and M5Tree, respectively, in Eastern Australia.

In the context of South Africa, persistent drought occurrences have been a challenge over the past four decades (Meza et al., 2021). Notable among these drought episodes is the severe 2015–2017 period, which significantly reduced water availability in the Western Cape and Eastern Cape provinces (Mahlalela, et al., 2020; Omar & Abiodun, 2020). This crisis led to the declaration of a national disaster in the Western Cape (Visser, 2018), resulting in substantial losses, especially in the agricultural sector (Pienaar & Boonzaaier, 2018), and the well-documented “day zero” in Cape Town, impacting around 3.7 million people (Sousa, et al., 2018; Burls, et al., 2019; Pascale, et al., 2020). Various factors contribute to drought in South Africa, including changing rainfall patterns attributed to shift jet streams and storm tracks (Mahlalela, et al., 2018; Sousa, et al., 2018), Hadley Cell expansion in the Southern Hemisphere (Burls et al., 2019), and ocean–atmosphere interactions (Chivangulula et al., 2023). Additionally, increased water demand due to increased urbanization, water mismanagement, and insufficient investment in water reservoir infrastructure and agriculture exacerbate the situation (Mahlalela et al., 2020; Schreiner, et al., 2018; Meza, et al., 2021). Continued climate change and global warming are anticipated to perpetuate the prevailing drought conditions in the forthcoming years (Abiodun, et al., 2018; Engelbrecht et al., 2009; Naik & Abiodun, 2019). Consequently, the predominant focus of drought mitigation responses in South Africa has been directed toward the agricultural sector (du Pisani, et al., 1998; Kamali, et al., 2018; Magombeyi & Taigbenu, 2008; Masupha & Moeletsi, 2020; Muyambo, et al., 2017; Schwarz et al., 2020), particularly at the national level. Notably, Nxumalo et al. (2022) highlighted the adverse impact of drought on wheat production in South Africa, especially in Free State and Mpumalanga provinces, resulting in significant reductions in production levels (Chikoore & Jury, 2021; Chivangulula, et al., 2023). Furthermore, Nemukula et al. (2023) employed Schlather model to analyze drought characteristics in the Lowveld region of Limpopo. Additionally, Naik and Abiodun (2019) used CORDEX models, obtaining correlations of 0.38 using the Standardized Precipitation-Evapotranspiration Index (SPEI) and 0.24 for SPI when comparing observed and simulated droughts. Mathivha et al. (2020) forecasted drought in the Vhembe District of Limpopo, utilizing Generalized Additive Models (GAM), Ensemble Empirical Mode Decomposition (EEMD)-GAM, EEMD-Autoregressive Integrated Moving Average (ARIMA)-GAM and fQRA models, each yielding different correlation ranges, namely 0.48 to 0.95 for GAM, 0.79 to 0.95 for EEMD-GAM, 0.94 to 0.99 for EEMD-ARIMA-GAM, and 0.92 to 0.99 for Forecast Quantile Regression Averaging (fQRA). Ikegwuoha and Dinka (2020) simulated drought in the Lepelle River Basin (LRB) in South Africa using the GCM, resulting in a correlation of 0.836 and predicting increased drought conditions in the twenty-second century. These studies show that different drought models performed differently, highlighting the complexity and uncertainty of drought modelling. Hence, the aim of this study is to create models for predicting meteorological drought over some selected locations in South Africa (Cape Town (CPT), Umtata (UMT), Spring Bok (SB), and Upington (UPT)) using Artificial Neural Network (ANN) and meteorological variables. This research will further identify the optimal parameter set for simulating meteorological drought in each location while utilizing SPI indices at 3-month and 6-month timescales.

## Study area, data, and methodology

### Study area

**Fig. 1:**
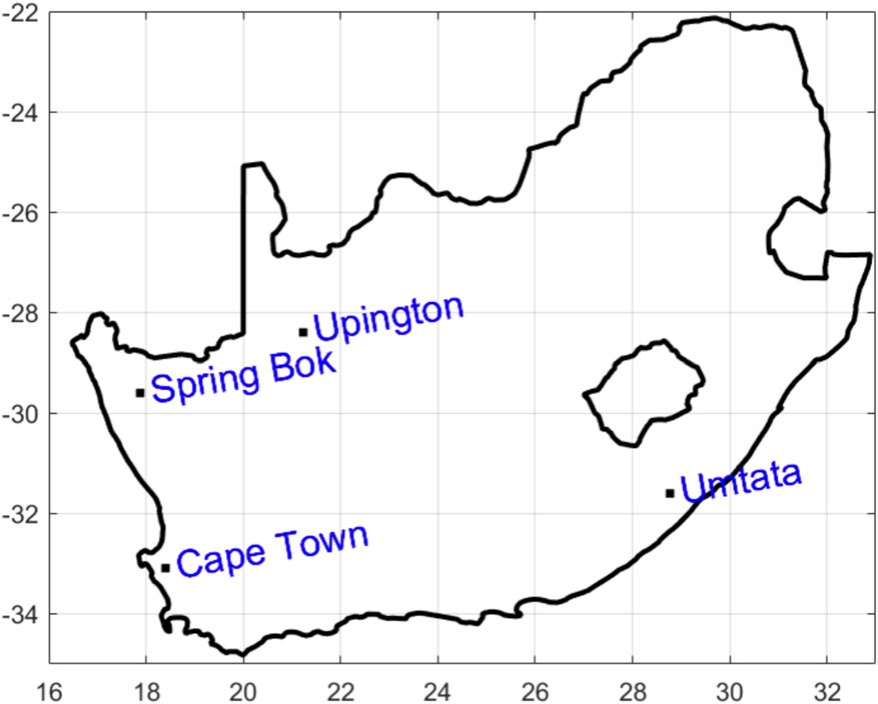
Map of study area showing the sample sites

South Africa spans latitudes 22° S to 35° S and longitudes 17° E to 33° E (refer to Fig. 1), situated in the southernmost part of Africa. Encompassing an area of approximately 1,219,602 km^2^, it boasts a coastline extending over 3000 km.

The country’s landscape encompasses diverse topography, featuring an arid desert in the northwestern region and somewhat arid conditions along the eastern coast. Positioned within the “drought belt” region of sub-Saharan Africa, South Africa contends with water constraints, akin to numerous neighboring nations. Rainfall duration varies across distinct geographical areas. In the Western Cape, the peak rainfall occurs during winter, contrasting with other regions that experience their highest precipitation in summer. On average, annual precipitation over South Africa totals approximately 464 mm (see Table 1 for average precipitation in each study location). Consequently, South Africa can be divided into two major physiographic regions: the interior plateau, and the land between the plateau and the coast separated by the Great Escarpment. The interior plateau is part of the great African plateau which stretches to the Sahara Desert (Webber, 2008; SAG, 2024). Also, temperature in South Africa is lower when compared with other countries at similar latitudes due to its high elevation. Summer temperatures typically range from 15 to 36 °C, while winter temperatures vary between − 2 and 26 °C. The interior plateau with altitude of 1694 m maintains an average summer temperature below 30 °C and a freezing night temperature during winter, while coastal regions are relatively warm during winter. Furthermore, there is a differential temperature gradient between east and west coasts; hence, the warm Agulhas Current sweep through the east and cold Benguela Current through the west coastlines, respectively (SAG, 2024).

**Table 1:** Geographical information of the four locations selected for this study

Location	Coordinate	Altitude ASL (m)	Climate
Cape Town	33.9° S, 18.42° E	1590.4	Semi-arid, warm Mediterranean climate, moderately wet winters, and dry warm summers. Yearly average precipitation 515 mm, relative humidity 76%
Spring Bok	29.6° S,17.88° E	950	Semi-arid, hot, and dry summer cold desert. Yearly average precipitation 215 mm, relative humidity 44%
Umtata	31.6° S, 28.77° E	698	Semi-arid, warm oceanic, humid subtropical climate, and semi-arid climate. Yearly average precipitation 650 mm, relative humidity 59%
Upington	28.4° S, 21.23° E	835	Arid, hot desert, hot summers, and short mild winters. Maximum Precipitation during late summer. Yearly average precipitation 189 mm, relative humidity 40%

On the other hand, north of South Africa lie Namibia, Botswana, and Zimbabwe, while Mozambique and Eswatini border its eastern side. Additionally, Lesotho is an enclave within South Africa. The Indian and Atlantic Oceans, respectively, are South Africa’s southern and western borders, whereas Cape Agulhas is the demarcation point where these two oceans intersect. For this study, the selected sites include Cape Town (CPT) in the Western Cape, Spring Bok (SB), and Upington (UPT) in the Northern Cape, as well as Umtata (UM) in the Eastern Cape (refer to Table 1).

### Data

The Modern-Era Retrospective Analysis for Research Version 2 (MERRA-2) data replaced the earlier MERRA resources, offering a comprehensive Earth System re-analysis dataset inclusive of meteorological and various climatic data sets. MERRA-2 is a culmination of integrated data from space-based instruments and reliable climate sources. For this study, we utilized meteorological data encompassing relative humidity, temperature, soil wetness, evapotranspiration, and evaporation with a spatial resolution of 0.5°. Soil wetness, evapotranspiration, and evaporation were acquired through NASA GIOVANNI (https://giovanni.gsfc.nasa.gov/giovanni/#service=TmAvMp&starttime=&endtime=&variableFacets=dataFieldMeasurement%3AEvaporation%2CEvapotranspiration%2CSoil%20Moisture%3BdataProductPlatformInstrument%3AMERRA-2%20Reanalysis%3B), while temperature and relative humidity were accessed from (https://power.larc.nasa.gov/data-access-viewer/).

The Moderate Resolution Imaging Spectroradiometer (MODIS) sensors are installed on both the Terra and Aqua satellites, orbiting the Earth twice daily: Terra in the morning (north to south) and Aqua in the afternoon (south to north). These instruments provide valuable insights into climate dynamics and processes globally, spanning land, ocean, and atmospheric regions. This study specifically employed MODIS Aqua Aerosol Optical Depth (AOD) and Sea Surface Temperature (SST) data at a spatial resolution of 0.1° (acquired from https://ladsweb.modaps.eosdis.nasa.gov/search/order/2/MCD19A2--6.). These data were set to the same grid as the MERRA 2 inputs.

All the data utilized in this study covered a temporal span of 21 years (from 2000 to 2021), and monthly averages were computed for analysis.

### Data integration

**Fig. 2:**
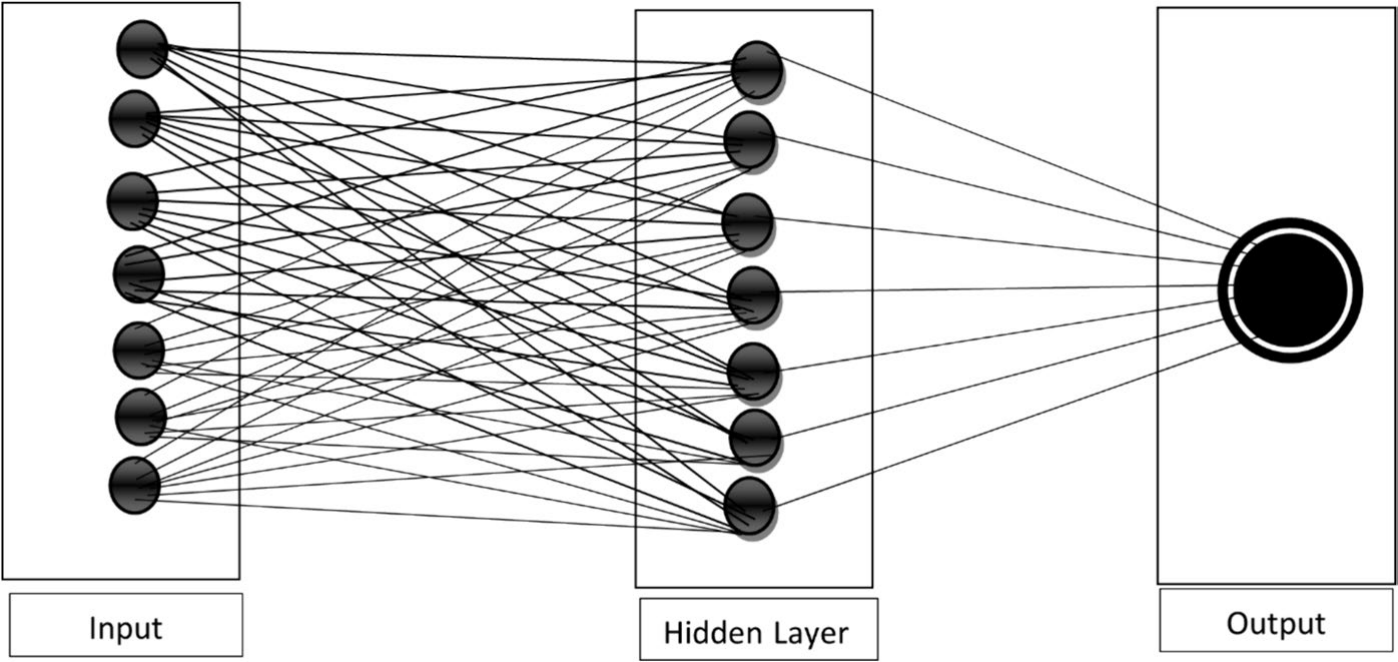
Sample schematic illustration of the neural network with Architecture 7–7-1 used in this study

To integrate all the datasets for model creation, an Artificial Neural Network (ANN) was employed. The ANN, a technique utilized across various fields, including drought studies (as mentioned in the introduction), has demonstrated its reliability as a statistical analysis tool. The specific architecture of the utilized ANN is structured as Input-Hidden Layer Neuron-Output (refer to Fig. 2). The input layers are the combinations of parameters, while the SPI value represents the target or output. Training the network involved employing the Levenberg‐Marquardt (LM) algorithm, selected for its efficiency in minimizing error functions and reducing training time during neural network training (Jang et al., 1997). Additionally, the MATLAB tansig function, employed to facilitate the transfer of functions from the input layer to the hidden layer and subsequently to the output, is given in Eqs. 1a and 1b (see also Okoh et al., 2019; Onyeuwaoma et al., 2021). Tansig function also introduces non-linearities into neural networks, enabling them to learn and model complex patterns and relationships within a set of data.

1a $${\text{H}}_{\text{m}}=\text{tanh}({\text{I}}_{\text{wm}}\times {\text{I}}_{\text{m}}+{\text{B}}_{1})$$

1b $${\text{O}}_{\text{m}}=\text{tanh}({\text{H}}_{\text{wm}}\times {\text{H}}_{\text{m}}+{\text{B}}_{2})$$

Equation 1a connects the input layer matrix ${\text{I}}_{\text{m}}$ to the hidden layer matrix ${\text{H}}_{\text{m}}$, and Eq. 1b connects the hidden layer matrix to the output layer matrix ${\text{O}}_{\text{m}}$. ${\text{I}}_{\text{m}}$ contains inputs for the neural network, ${\text{H}}_{\text{m}}$ contains intermediary values computed within the hidden layer, and ${\text{O}}_{\text{m}}$ contains outputs from the neural network. I_wm_ and H_wm_ are the respective weight matrices for the input and the hidden layers. B_1_ and B_2_ are the bias vectors for the input and the hidden layers. The input weight matrices and the bias vectors contain constants for a given trained neural network. 

Subsequently, 70% of the collected data was allocated for training, while 15% each was set aside for validation and another 15% for testing purposes. This data allocation formula had been applied in several research as well. Determining the optimal number of hidden layer neurons (HLN) for each model involved applying a method developed by Dan Okoh (refer to Okoh, 2023). This method entailed generating 100 networks for each model (refer to Table 2 for the data combinations utilized in these models). The number corresponding to the network with the lowest root mean square error (rmse) was selected as the input value for the HLN count. The input datasets are relative humidity (rh), temperature (tp), evapotranspiration (et), evaporation (ev), soil wetness (sw), sea surface temperature (st), and aerosol optical depth (aa). These parameters chosen have a link with drought conditions such that at low RH, the rate of transpiration is on the increase especially during drought. Consequently, droughts are also associated with clear sky that ensures an increase in incoming solar radiation and subsequent higher daytime temperatures. Further to this, rise in evapotranspiration (et) is an important trigger factor for seasonal drought in any given region; hence, it increases during drought. Also, warmer temperatures enhance evaporation (ev), which reduces surface water and dries out soils and vegetation. Hence, abnormal dryness of soil is associated with drought condition. This makes the period of low precipitation drier than it would be during cooler conditions. Sea surface temperature (SST), on the other hand, gives information on the heat content at the ocean surface. SST influences the earth’s climate (Gonsamo et al., 2016), through variations in climate factors (such as temperature, soil moisture, precipitation) which are associated with drought (see Yan et al., 2019; Kumar et al., 2024). Atmospheric aerosols measured as aerosol optical depth (aa) influence the hydrological cycle in the atmosphere depending on their optical properties. Some species of aerosol scavenge for the available water vapor thereby exacerbating the dry condition. Furthermore, drought conditions increase the emission of aerosols into the atmosphere especially dust-related species.

**Table 2:** Set combinations used to generate the simulations

Set simulations	Models
1	rh-tp-sw-st-ev-st-aod-et
2	rh-tp
3	rh-tp-sw
4	rh-tp-sw-et
5	rh-tp-sw-ev
6	rh-tp-sw-st
7	rh-tp-sw-st-et
8	rh-tp-sw-st-ev
9	tp-sw
10	tp-sw-et
11	tp-sw-ev

For the analysis, data spanning from 2000 to 2017 were employed to train the network, while the remaining (from 2018 to 2021) were reserved for validating the models.

Furthermore, analysis of variance test (ANOVA) was used to statistically analyze the differences between simulated and measured SPIs. The values of the *F*-statistic and *p*-value (0.05) determine when to accept or reject the null hypothesis.

### Standard precipitation index (SPI)

Monitoring and prediction of drought often rely on indices such as the Standard Precipitation Index (SPI) ( see Mckee et al., 1993; Karavitis et al., 2011), Standardized Streamflow Index (SSI) (see Telesca et al., 2012; Shamshirband et al., 2020), Standardized Precipitation-Evapotranspiration Index (SPEI) (see Vicente-Serrano et al., 2010; Tirivarombo et al., 2018), Percent of Normal Precipitation (PNP) ( see Amiri & Gocic, 2023), and Palmer Drought Severity Index (PDSI) (see: Dai, 2011; Yu et al., 2019).

The SPI quantifies precipitation deficiencies across varying time frames. This study focuses on meteorological drought utilizing the SPI, recommended by the World Meteorological Organization (WMO) due to its versatility in assessing drought over periods ranging from 3 to 24 months (Mckee et al., 1993).

The SPI, which is a function of probability density function for the gamma distribution *g*(*x*), is given as:

2 $$g\left(x\right)=\frac{1}{\beta \gamma (\alpha )}{x}^{\alpha -1}{e}^{-x/\beta }$$

where $\propto >0$ is the shape parameter, $\beta >0$ is the scale parameter, and *x* is the rainfall measurement. The gamma function $\gamma (\alpha )$ (University of Arizona, 2023) shown in the above equation is defined as

3 $$\gamma \left(\alpha \right)=\underset{0}{\overset{\infty }{\int }}{y}^{\propto -1} {e}^{-y} dy$$

4 $$\widehat{\propto }=\frac{1}{4A} \left(1+\sqrt{1+\frac{4A}{3}}\right)$$

5 $$\widehat{\beta }=\frac{\overline{x}}{\widehat{\alpha } }$$

while,

6 $$A=\text{ln}\overline{x }-\frac{\sum \text{ln}(x)}{n}$$

where *n* is the number of rainfall measurements, and $\overline{x }$ is the mean of *x.*

The SPI was categorized into distinct events based on the computed values, detailed in Table 3. Following this schema, a drought event initiates when the SPI values dip below zero and concludes when they rise to a positive range.

**Table 3:** Drought intensity categories calculated from SPI (McKee et al. (1993))

SPI values	Drought category	Time in category
0 to − 0.99	Mild drought	~ 24%
− 1.00 to − 1.49	Moderate drought	9.2%
− 1.50 to − 1.99	Severe drought	4.4%
≤ − 2.00	Extreme drought	2.3%
		~ 40%

## Result and discussion

### Time series plots for SPI 3 and SPI 6

**Fig. 3:**
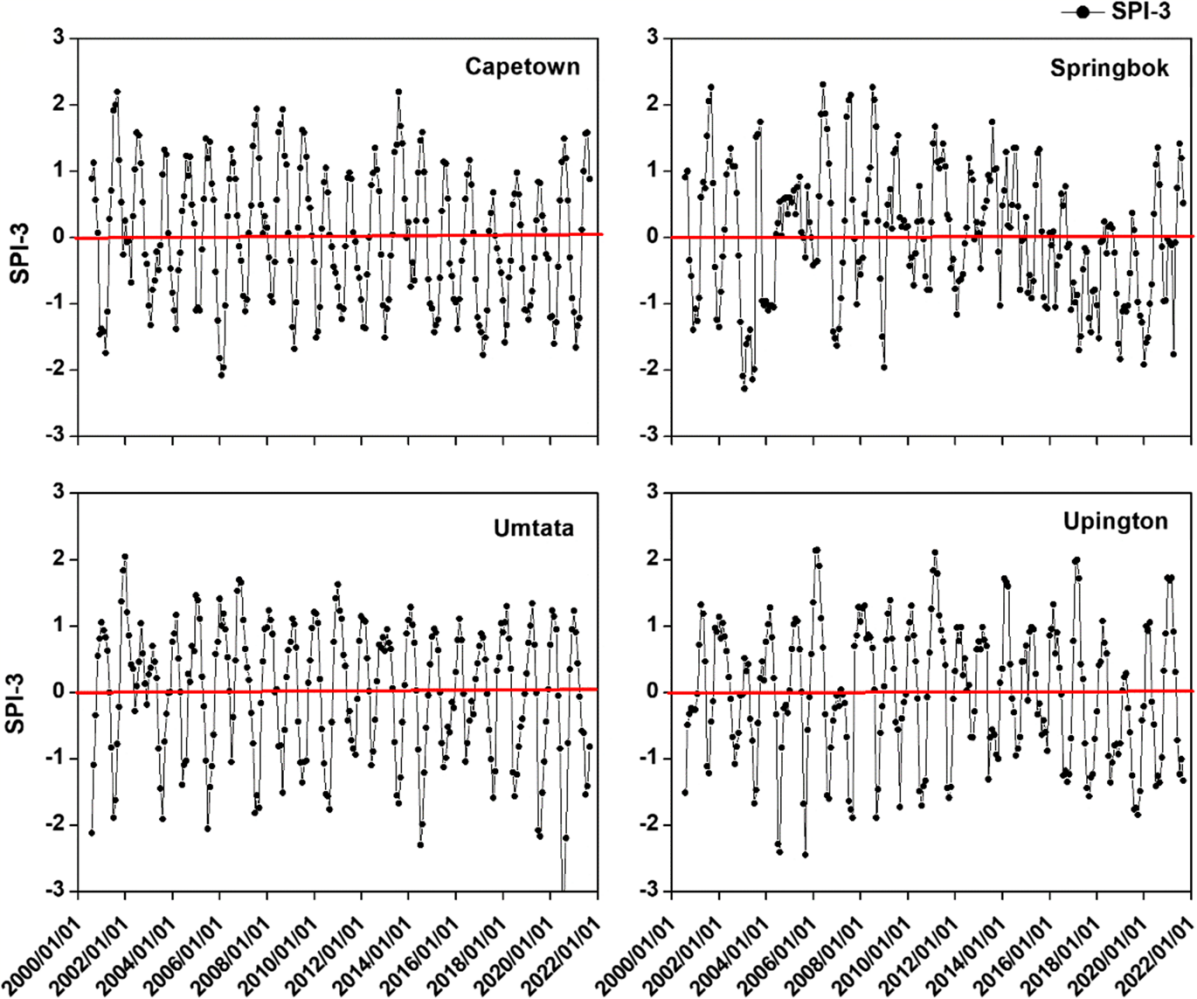
SPI3 time series for the study period

The primary objective of this study is to compute the Standardized Precipitation Index (SPI) for four specific locations at 3-month (SPI 3) and 6-month (SPI 6) timescales, detailed in Fig. 3 and Table 4 which are the timeseries plots and classification of droughts derived from calculating the SPI using Eq. 2.

**Table 4:** Percentage of occurrence of the different drought categories for study period

Drought category	Cape Town		Spring Bok		Umtata		Upington	
	SPI 3 (%)	SPI 6 (%)	SPI 3 (%)	SPI 6 (%)	SPI 3 (%)	SPI 6 (%)	SPI 3 (%)	SPI 6 (%)
Mild drought	27.56	29.08	30.31	29.88	24.8	21.91	31.5	30.68
Moderate drought	17.72	13.55	12.6	9.56	9.84	12.35	11.42	11.95
Severe drought	4.33	3.59	5.91	5.98	6.69	5.98	6.3	4.38
Extreme drought	0.39	1.99	1.18	2.39	3.15	2.79	1.18	1.99
Extreme wet	1.18	0.8	2.76	1.2	0.39	0	1.57	1.2
Very wet	5.52	5.18	4.72	5.58	1.98	4.38	4.33	5.18
Moderately wet	12.99	11.95	10.63	8.76	14.57	12.35	9.84	10.36
Mild wet	30.31	33.86	31.89	36.65	38.58	40.24	33.86	34.26

**Fig. 4:**
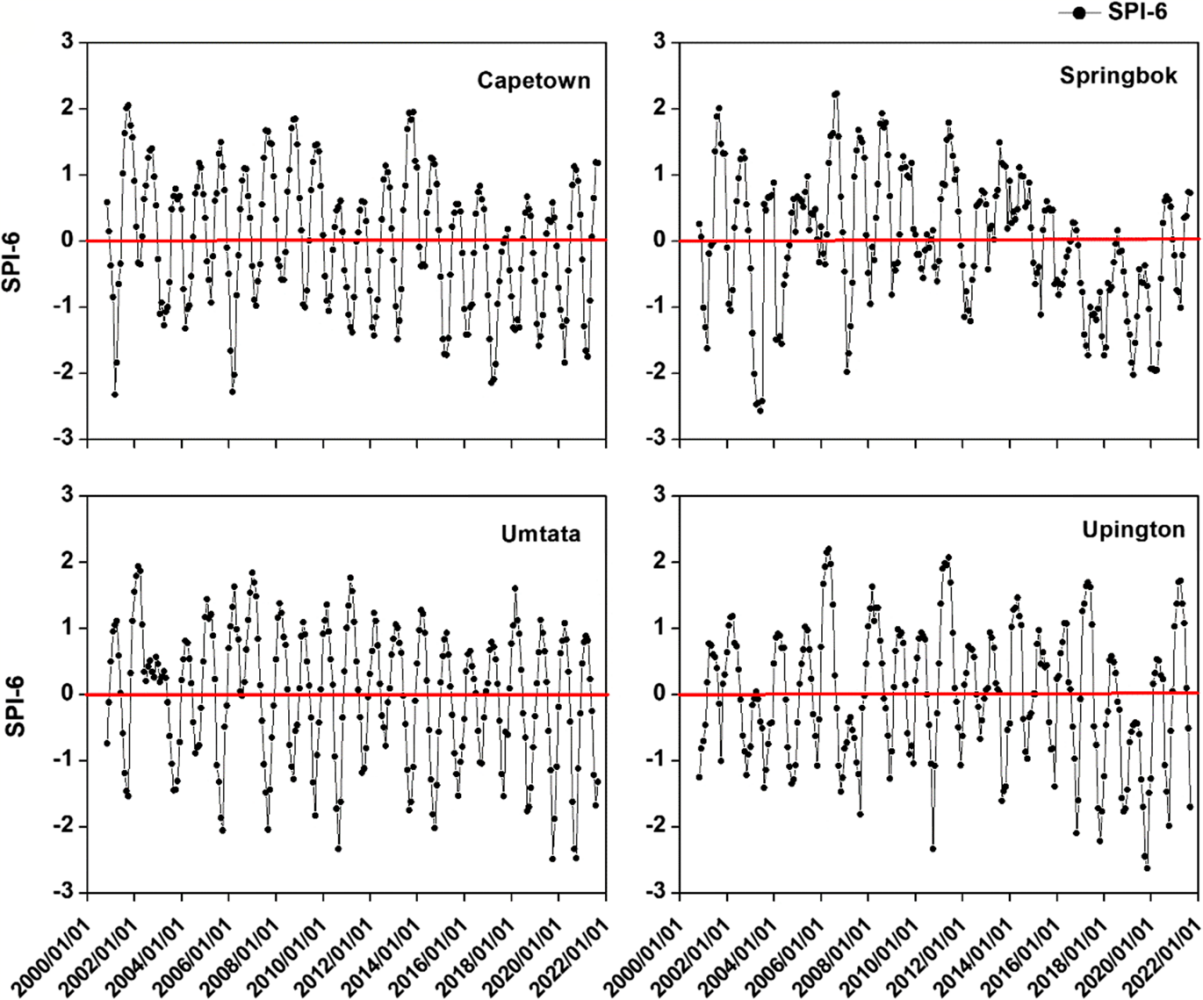
SPI6 time series for the study period

Figure 3 displays the findings indicating sustained drought across all locations during the specified period. The most severe drought was observed at Spring Bok from 2016 to 2020, followed by Cape Town during the same timeframe. Examining Fig. 4 reveals an escalating intensity of drought, notably noticeable at Spring Bok (SB) and Cape Town (CPT) between 2013 and 2021 for SB, 2014 and 2020 for CPT, and 2018 and 2021 at UMT. The data in Table 4 demonstrates the percentage of SPI 3 drought occurrences, indicating that mild drought was the predominant category across all locations: CPT (27.56%), SB (30.31%), UMT (24.8%), and UPT (31.5%). This was followed by moderate drought. Notably, the most severe drought conditions were recorded at UMT (9.84%), whereas SB experienced the highest proportion of extreme wetness (2.76%). Analyzing the different drought categories for SPI 6, as presented in Table 4, reveals the prevalence of mild drought across all locations, with 30.68% at UPT and 29.88% at SB. The highest percentage of the most critical drought conditions occurred at UM (8.77%) and SB (8.37%).

Further examination of Table 4 reveals that for SPI3, Cape town (CPT) experienced drought conditions 50% of the time and was wet for the remaining 50%, similar to Spring Bok (SB) with 44.48% drought and 55.52% wet, and UPT 50.4% drought and 49.6% wet. Looking at SPI 6, CPT encountered drought 48.21% of the time and was wet for 51.79%, while SB had 47.81% drought and 52.19% wet; UM experienced 43.03% drought and 56.97% wet, and UPT faced 49% drought and 51% wet conditions. This analysis suggests that short-term drought situations occur more frequently in CPT and SB, while longer-lasting drought conditions are more prevalent in UPT and CPT. The shift of the Inter-tropical Convergence Zone (ITCZ) southwards due to the warming of the Atlantic Ocean is similar to observed patterns over the Sahel region (Epule et al., 2014). The result is a decrease in moisture input from the Ocean and weakening of the West African Monsoon (WAM) which creates drier conditions, vegetation losses, and increased surface albedo ( seeZeng, 2003; Bader & Latif, 2003; Zhang & Delworth, 2006). This could potentially account for the drought patterns observed around UPT, CPT, and SB, which are located along the Atlantic coast.

### ANN model results

In this section, we examined the performance of the 11 distinct Artificial Neural Network (ANN) configurations using various sets of parameters to develop models for each location studied across different SPI timescales. Following the analysis, we determined the most effective model for each specific station.

**Fig. 5:**
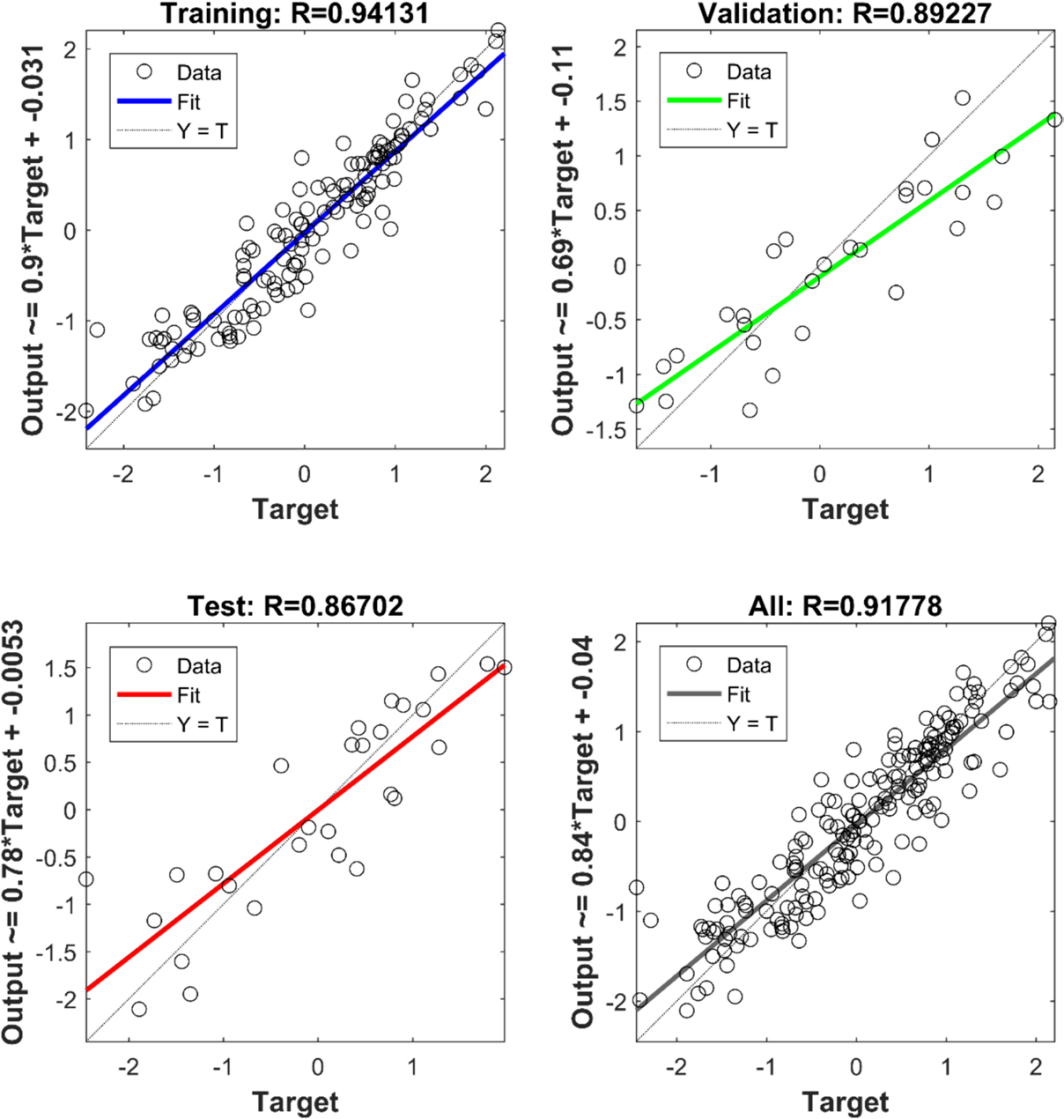
Sample statistic of the input combinations for forecasting of SPI, showing the relationship between the output and target

Figure 5 and Table 5 present the statistical analysis of the Artificial Neural Network (ANN) models from the different sets of parameters considered. In Fig. 5, a visual representation illustrates how the dataset performed concerning the independent variable output. The dashed line represents the ideal scenario, while the solid line reflects the actual performance, and the gap between them signifies the network’s deviation from the ideal situation. In this instance, the *R* values indicate strong performance, consistently noted ~ 0.8 or higher. The remaining statistics of the network is detailed in Table 5. The table indicates that for SPI 3, the *R* values across all locations range from 0.71 to 0.99, with mean square error (MSE) values varying between 0.04 and 0.6 across different data divisions (such as training, validation, and testing). For SPI 6, the *R* value range from 0.50 to 0.99, and MSE ranges from 0.09 to 0.99. These findings suggest that the models perform better in predicting SPI 3 compared to SPI 6. Moreover, Table 5 highlights that the ANN training results exhibited comparatively lower performance at Spring Bok. Notably, in the analysis conducted at Cape Town, sets 6, 7, and 8 were excluded due to some persistent errors encountered in the models.

**Table 5:** Statistical details of input combinations utilized for forecasting SPI through an Artificial Neural Network (ANN). The measurement includes mean square error (MSE) and coefficient of determination (*R*) specifically for CPT and SB, also NR implies no result as earlier specified

Sim. Set		CPT				SB				UMT				UPT			
		SPI3		SPI6		SPI3		SPI6		SPI3		SPI6		SPI3		SPI6	
		MSE	*R*	MSE	*R*	MSE	*R*	MSE	*R*	MSE	*R*	MSE	R	MSE	R	MSE	*R*
1	Training	0.1	0.96	0.09	0.95	0.31	0.81	0.16	0.92	0.049	0.97	0.17	0.92	0.13	0.93	0.13	0.92
	Validation	0.3	0.82	0.11	0.94	0.63	0.7	0.13	0.5	0.14	0.91	0.17	0.9	0.13	0.93	0.24	0.89
	Testing	0.11	0.95	0.24	0.88	0.44	0.89	0.46	0.66	0.16	0.92	0.36	0.8	0.37	0.81	0.4	0.8
2	Training	0.27	0.85	0.79	0.43	0.49	0.73	0.68	0.56	0.18	0.9	0.61	0.56	0.34	0.82	0.3	0.8
	Validation	0.44	0.81	0.62	0.57	0.51	0.73	0.52	0.7	0.13	0.9	0.46	0.74	0.31	0.81	0.47	0.78
	Testing	0.44	0.71	0.88	0.52	0.39	0.7	0.99	0.64	0.33	0.81	0.82	0.52	0.45	0.73	0.41	0.77
3	Training	0.18	0.91	0.4	0.78	0.32	0.82	0.34	0.82	0.65	0.96	0.51	0.64	0.3	0.82	0.34	0.81
	Validation	0.11	0.94	0.48	0.7	0.29	0.84	0.61	0.6	0.38	0.84	0.41	0.75	0.39	0.81	0.4	0.7
	Testing	0.29	0.84	0.56	0.6	0.5	073	0.73	0.55	0.2	0.86	0.67	0.67	0.24	0.86	0.29	0.79
4	Training	0.2	0.91	0.15	0.91	0.29	0.83	0.29	0.82	0.063	0.96	0.33	0.81	0.18	0.9	0.11	0.94
	Validation	0.16	0.92	0.21	0.91	0.62	0.75	0.3	0.83	0.22	0.9	0.29	0.79	0.18	0.92	0.45	0.77
	Testing	0.12	0.92	0.35	0.86	0.34	0.77	0.72	0.65	0.15	0.92	0.16	0.94	0.43	0.83	0.48	0.78
5	Training	0.32	0.99	0.38	0.99	0.36	0.81	0.41	0.74	0.12	0.92	0.11	0.93	0.15	0.92	0.17	0.89
	Validation	0.4	0.83	0.32	0.84	0.25	0.78	0.49	0.77	0.11	0.94	0.35	0.81	0.24	0.89	0.32	0.85
	Testing	0.44	0.76	0.54	0.76	0.36	0.82	0.42	0.75	0.14	0.93	0.24	0.87	0.37	0.85	0.23	0.9
6	Training	NR	NR	NR	NR	0.35	0.81	0.24	0.85	0.09	0.95	0.51	0.67	0.15	0.91	0.3	0.82
	Validation	NR	NR	NR	NR	0.32	0.86	0.94	0.65	0.16	0.91	0.66	0.72	0.26	0.9	0.32	0.83
	Testing	NR	NR	NR	NR	0.36	0.82	0.62	0.52	0.13	0.93	0.59	0.55	0.29	0.84	0.3	0.81
7	Training	NR	NR	NR	NR	0.36	0.81	0.35	0.78	0.11	0.96	0.34	0.81	0.2	0.86	0.24	0.86
	Validation	NR	NR	NR	NR	0.27	0.82	0.6	0.73	0.45	0.81	0.49	0.66	0.19	0.88	0.2	0.85
	Testing	NR	NR	NR	NR	0.29	0.84	0.39	0.8	0.36	0.76	0.43	0.72	0.4	0.82	0.28	0.87
8	Training	NR	NR	NR	NR	0.36	0.75	0.23	0.87	0.1	0.95	0.14	0.92	0.23	0.88	0.25	0.85
	Validation	NR	NR	NR	NR	0.34	0.88	0.16	0.66	0.22	0.87	0.29	0.81	0.37	0.8	0.3	0.77
	Testing	NR	NR	NR	NR	0.31	0.87	0.68	0.58	0.14	0.94	0.34	0.83	0.51	0.79	0.46	0.81
9	Training	0.13	0.93	0.5	0.71	0.28	0.83	0.27	0.83	0.16	0.9	0.52	0.65	0.36	0.79	0.34	0.81
	Validation	0.18	0.91	0.52	0.71	0.42	0.81	0.98	0.52	0.6	0.71	0.82	0.64	0.34	0.82	0.4	0.77
	Testing	0.29	0.83	0.67	0.56	0.49	0.82	0.74	0.66	0.51	0.82	0.41	0.77	0.47	0.77	0.24	0.8
10	Training	0.12	0.94	0.18	0.9	0.28	0.83	0.37	0.78	0.95	0.94	0.43	0.73	0.26	0.86	0.24	0.84
	Validation	0.15	0.92	0.27	0.87	0.44	0.82	0.64	0.7	0.27	0.83	0.4	0.75	0.15	0.9	0.34	0.84
	Testing	0.13	0.92	0.33	0.86	0.54	0.76	0.73	0.61	0.18	0.91	0.74	0.55	0.21	0.9	0.25	0.88
11	Training	0.13	0.93	0.21	0.9	0.32	0.84	0.26	0.84	0.98	0.94	0.4	0.74	0.37	0.8	0.26	0.84
	Validation	0.16	0.92	0.25	0.88	0.38	0.81	0.75	0.56	0.13	0.93	0.28	0.82	0.23	0.83	0.29	0.84
	Testing	0.27	0.87	0.31	0.84	0.64	0.74	0.71	0.61	0.25	0.87	0.52	0.74	0.4	0.79	0.32	0.84

Typically, the relationship between *R* and MSE ($R\propto \frac{1}{MSE}$) holds across all locations. Consistently, the training, validation, and testing results follow a similar trend at Cape Town (CPT), Umtata (UMT), and Upington (UPT), except for Spring Bok (SB). At SB, there are instances where either R or MSE is notably high for specific data divisions, as depicted in Figs. 15 and 16 in the Appendix.

**Fig. 6:**
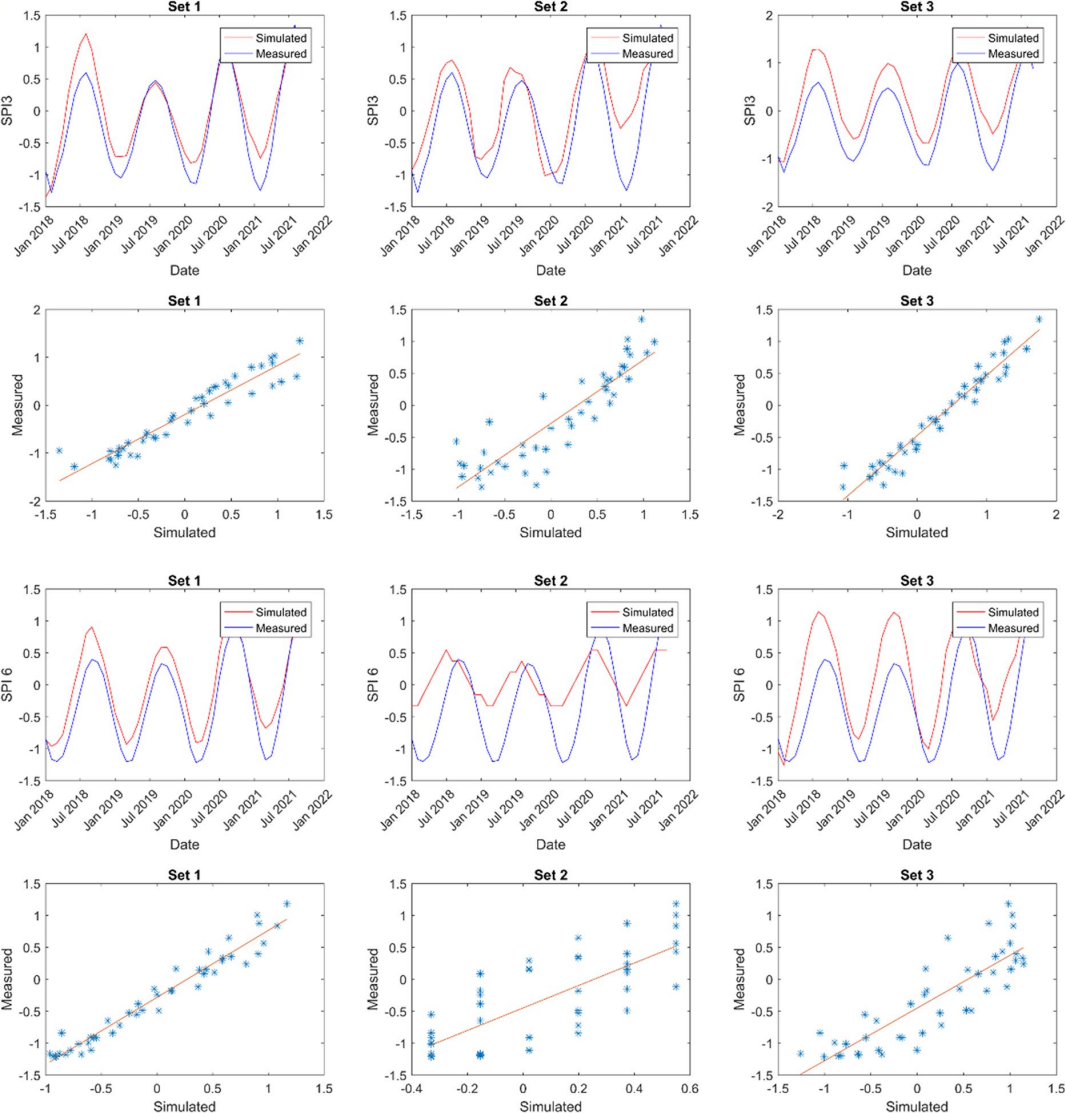
Sample of model results for Cape Town showing SPI 3 and SPI 6 time series alongside scatter plots for comparison

**Fig. 7:**
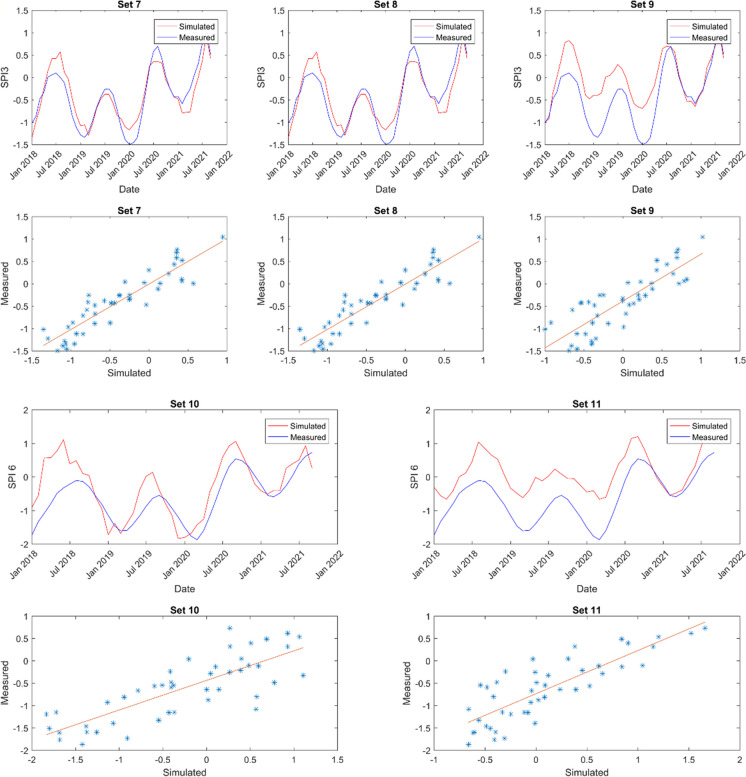
Sample of model results for Spring Bok showing SPI 3 and SPI 6 time series alongside scatter plots for comparison

**Fig. 8:**
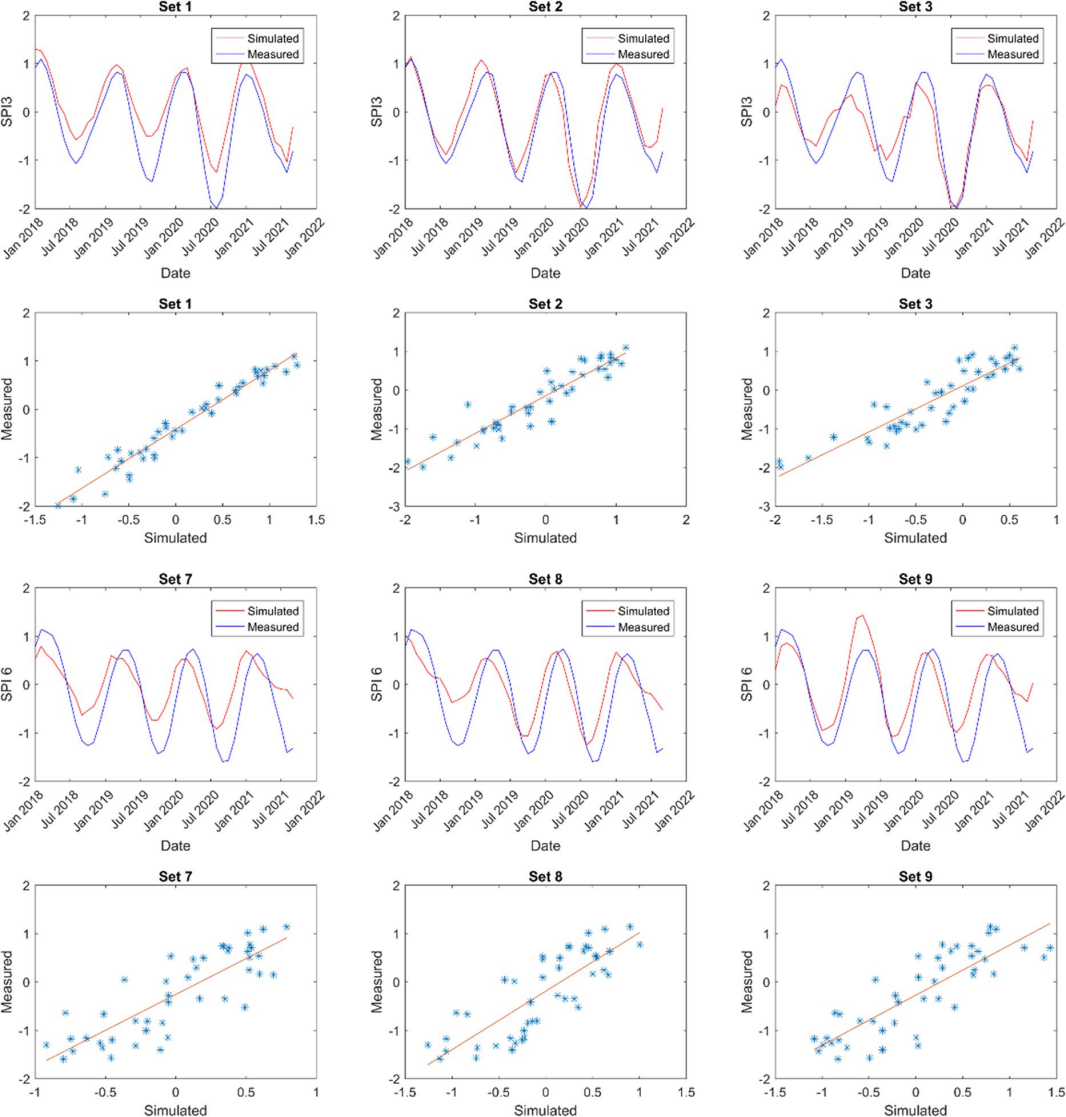
Sample of model results for Umtata showing SPI 3 and SPI 6 time series and scatter plots for comparison

**Fig. 9:**
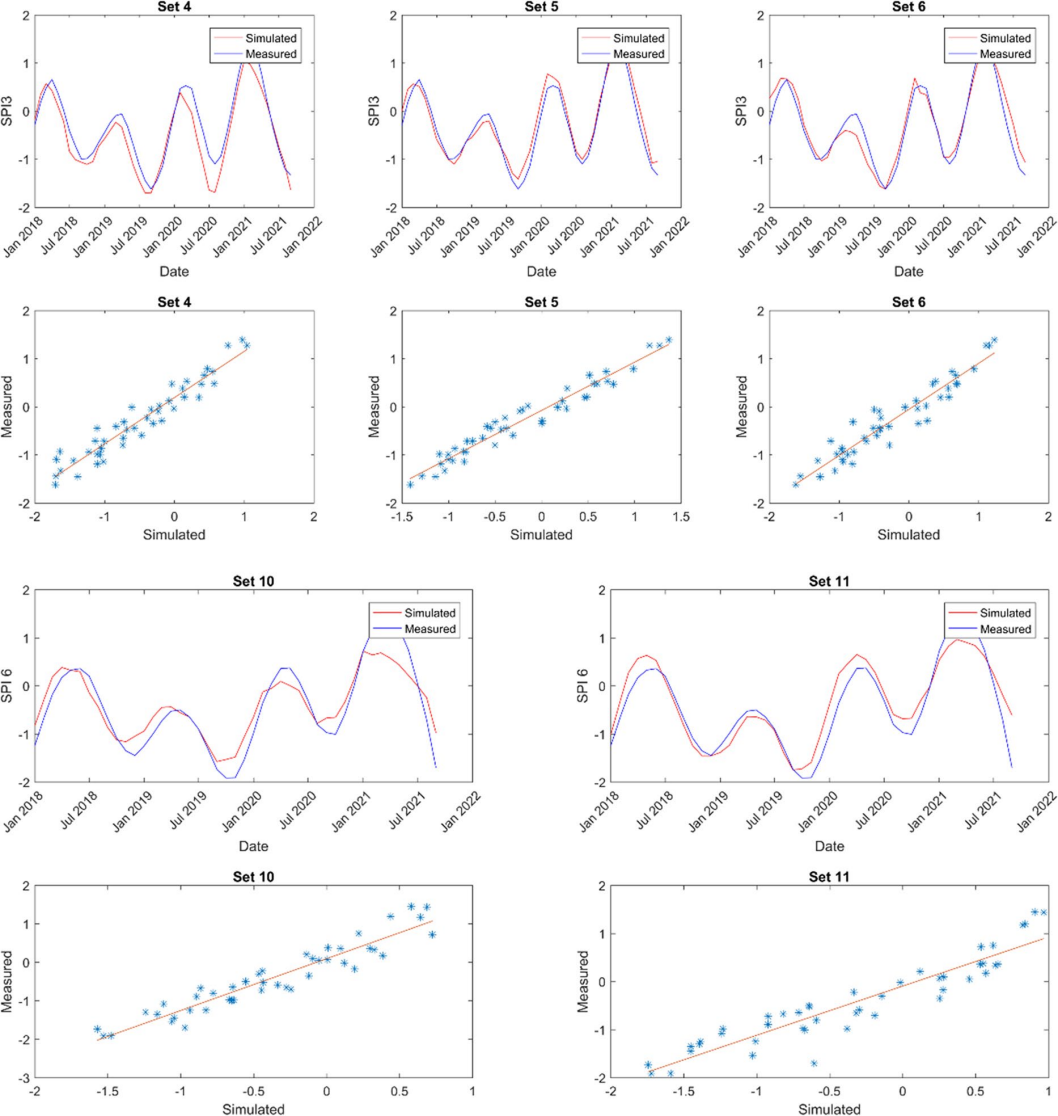
Sample of model results for Upington showing SPI 3 and SPI 6 time series and scatter plots for comparison

The model predictions’ outcomes are showcased in Figs. 6, 7, 8, and 9. These displayed results serve illustrative purposes and may not represent the best outcomes for all locations. Consequently, additional graphs are not presented here to avoid the repetition, it is provided in the Appendix.

Figures 6 (also Figs. 16, 17, 18, and 19 in the Appendix for the remaining comparison plots for CPT) and 10 (also Table 9 in the Appendix) represent the model results for Cape Town (CPT), highlighting correlations ranging from 0.78 to 0.94 for SPI 3 and from 0.57 to 0.95 for SPI 6. Among these, the combination of set3 yielded the strongest correlation of 0.94 for SPI 3, whereas set2 and set5 produced the weakest correlations. For a longer-term prediction (SPI 6), set1 demonstrated the highest correlation of 0.95. Furthermore, the findings suggest that evapotranspiration (et) serves as a superior substitute for evaporation (ev) at both time scales. Notably, utilizing relative humidity (rh) and total precipitation (tp) exclusively does not yield favorable outputs compared to other parameters. Additionally, soil moisture (sw) emerges as a crucial parameter in drought modelling at this specific location. Furthermore, in Tables 6, the *p*-values are used to ascertain the validity/accuracy level of the simulations at 0.05 confidence level. Hence, in addition to the high correlation values, the *p*-values should be ≤ 0.05 for the result to be significant.

**Table 6:** Comparison with some previous studies on SPI and SPEI modelling

Model	Index	Study area	*R*^2^	Author
RFVL–HGS	SPI-3	Bangladesh (arid to semi-arid)	0.81	Adnan et al., 2021
RFVL–HGS	SPI-6	Bangladesh (arid to semi-arid)	0.82	Adnan et al., 2021
MLP ANN	SPI-3	Iran (arid)	0.89	Jalalkamali et al., 2015
ANFIS	SPI-6	Iran (arid)	0.88	Jalalkamali et al., 2015
WNN	SPI-3	Ethiopia (arid to semi-arid)	0.88	Belayneh et al., 2016
ICA-RSVR	SPI-3	Iran (semi-arid)	0.22	Hosseini-Moghari et al., 2017
ICA-RSVR	SPI-6	Iran (semi-arid)	0.40	Hosseini-Moghari et al., 2017
MARS	SPI	Australia (arid to semi-arid)	(0.99–0.98)	Deo et al., 2017
LSSVM	SPI	Australia (arid to semi-arid)	(0.94–0.98)	Deo et al., 2017
M5Tree	SPI	Australia (arid to semi-arid)	(0.91–0.98)	Deo et al., 2017
APARCH	SPI-12	Chéliff–Zahrez basin (Algeria). Semi-arid	(0.80 to 0.95)	Habibia et al. (2018)
Neuro-fuzzy	SPI-3	Iran (arid)	0.50	Rafiei-Sardooi et al., 2018
ARIMA	SPI-3	Iran (arid)	0.20	Rafiei-Sardooi et al., 2018
CORDEX	SPI	South Africa (semi-arid)	0.24	Naik & Abiodun, 2019
CORDEX	SPEI	South Africa (semi-arid)	0.38	Naik & Abiodun, 2019
ANFIS-PSO	SPI-3	Iran (semi-arid)	0.87	Kisi et al., 2019
ANFIS-PSO	SPI-6	Iran (semi-arid)	0.92	Kisi et al., 2019
SVM	SPEI (6)	Pakistan (Rabi) (semi-arid)	(0.95–0.97)	Khan et al., 2020
ANN	SPEI (6)	Pakistan (Rabi) (semi-arid)	(0.87–0.91)	Khan et al., 2020
KNN	SPEI (6)	Pakistan (Rabi) (semi-arid)	(0.65–0.86)	Khan et al., 2020
GAM	SPEI	South Africa (semi-arid)	(0.48–0.95)	Mathivha et al., 2020
EEMD-GAM	SPEI	South Africa (semi-arid)	(0.79–0.95)	Mathivha et al., 2020
EEMD-ARIMA-GAM	SPEI	South Africa (semi-arid)	(0.94–0.99)	Mathivha et al., 2020
fQRA	SPEI	South Africa (semi-arid)	(0.92–0.99)	Mathivha et al., 2020
SVM	SPEI (3)	Pakistan (Rabi) (semi-arid)	(0.69–0.94)	Khan et al., 2020
ANN	SPEI (3)	Pakistan (Rabi) (semi-arid)	(0.76–0.79)	Khan et al., 2020
KNN	SPEI (3)	Pakistan (Rabi) (semi-arid)	(0.60–0.71)	Khan et al., 2020
LSTM	SPI-6	Iran (semi-arid)	(0.88–0.90)	Gorgij et al., 2022
LSTM	SPI-3	Iran (semi-arid)	(0.83–0.85)	Gorgij et al., 2022
CNN-LSTM	SPIE-6	Turkey (semi-arid)	(0.68–0.75)	Mehr et al., 2023

In Spring Bok, illustrated in Figs. 7 (also Figs. 20, 21, 22, 23,  24, and 25 in the Appendix for the remaining comparison plots for SB) and 10 (Table 10 in the appendix), the results revealed that most models exhibited poor performance, displaying correlations ranging from 0.49 to 0.84 for SPI3 and from 0.22 to 0.72 for SPI6. Among the simulations tested for SPI3, set4, set7, and set8 demonstrated the most effective performance, achieving a correlation value of 0.84 each. Conversely, set2 exhibited the weakest correlation at 0.49. Specifically, for SPI6, set7 simulations showed the strongest correlation (0.72), while set2 recorded the least correlation at 0.22 followed by set8 (0.37). In most cases, there appears to be a consistence in the set performance at both timescales, suggesting the effective modelling of both timescales using the same dataset. Additionally, evapotranspiration (et) emerged as a critical parameter in these models. For effective drought simulation at this location, the best sets to use are 4, 7, 8, and 10 for SPI3 and sets 1 and 7 for SPI 6.

The results obtained from Umtata, presented in Figs. 8 (also Figs. 25, 26, 27, 28, 29, and 30 in the Appendix for the remaining comparison plots for UMT) and 10 (see also Table 11 in the Appendix), depicted model correlations ranging between 0.82 and 0.95 for SPI3, and between 0.61 and 0.88 for SPI6. Notably, for both SPI3 and SPI6, set1 resulted in the most favorable outputs, yielding corrections of 0.95 and 0.88, respectively. Conversely, set3 demonstrated the weakest at 0.82 for SPI3, and set2 exhibited the lowest correlation of 0.0 for SPI6. Furthermore, it was observed that incorporating evapotranspiration (et) enhanced simulation performance especially for SPI3.

In Fig. 9 (also Figs. 31, 32, 33, 34, 35, and 36 in the Appendix for the remaining comparison plots for UPT) and e (see also Table 12 in the Appendix), the findings for Upington revealed that simulation sets 5 and 10 yielded the most favorable outcomes for SPI3, while set2 (0.92) produced the best results for SPI 6. Conversely, the weakest correlations were observed with set9 (0.77) for SPI3 and set8 (0.78) for SPI6. Notably, the ANN exhibited more accurate drought predictions at both SPI3 and SPI6 in Upington compared to other locations studied. Additionally, the inclusion of both evapotranspiration (et) and evaporation (ev) consistently enhanced model performance when integrated into any parameter combination.

The behavior and results observed across the different locations indicate that drought modelling is influenced by a complexity of events and exhibits a high level of localization.

**Fig. 10:**
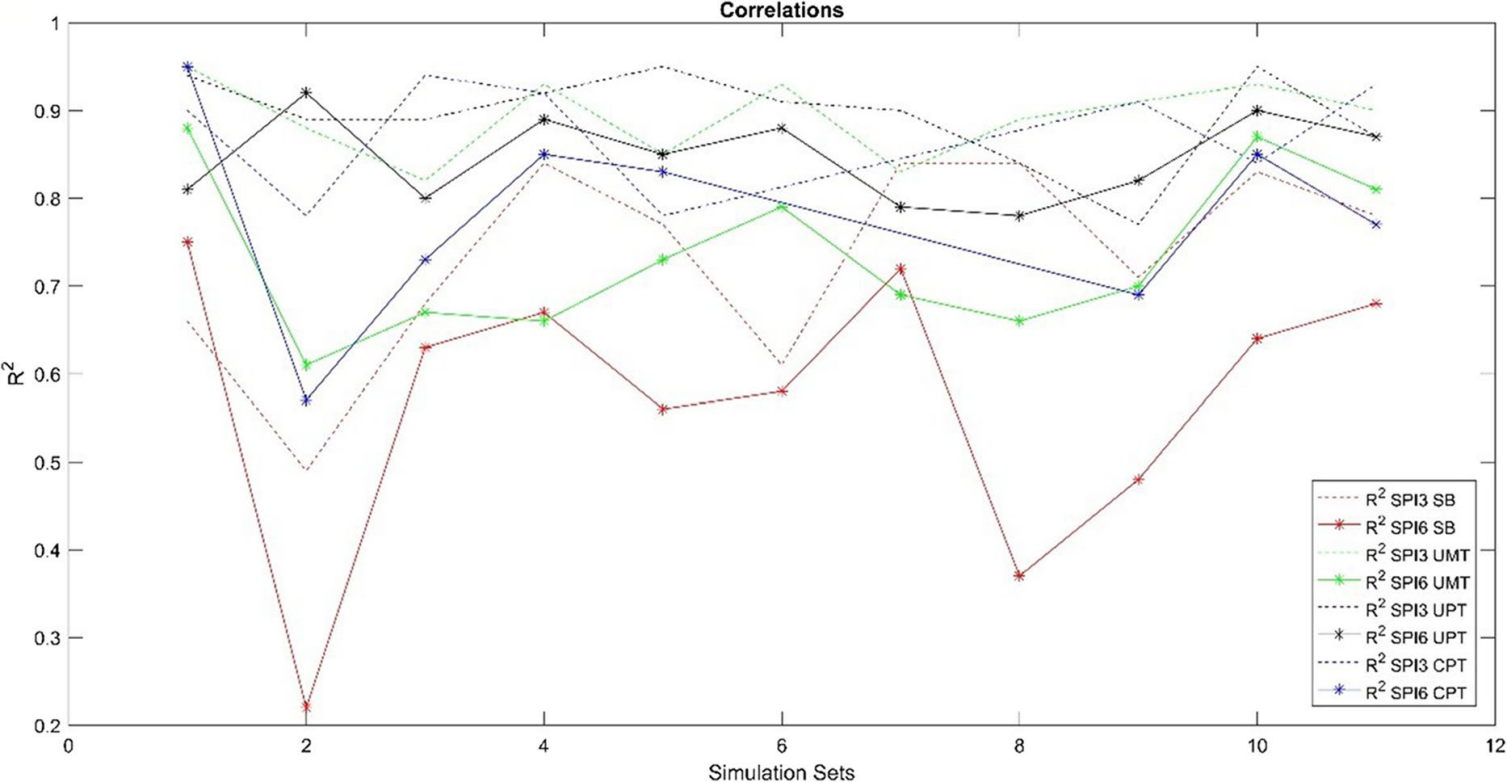
Correlation values of the different simulation sets at the different locations

In general, the results obtained using ANN indicate that most combinations of parameters exhibit strong predictive capabilities for drought at SPI3, while specific combinations perform well for SPI6. Sequel to this, the low *p*-values at most locations indicate that the set combinations can effectively simulate drought at both time scales; hence, they are below 0.05 confidence level, with the exception of some simulations at Spring Bok which proved otherwise (set2 (7.32e-08) for SPI3 and set2 (0.00127) and set9 (1.53e-07) for SPI 6). Therefore, the higher the *R*^2^ the lower the *p*-value. Across both timescales, it was consistently observed that the combinations demonstrating the strongest correlations tend to include evapotranspiration (et) and/or evaporation (ev) components. Noteworthy correlations of 0.95 for SPI3 were achieved at Umtata (UMT) (set1) and Upington (UPT) (set5 and set10), while the highest correlation for SPI6 (0.95) was attained with set1 at CPT. These outcomes are consistent with correlations found in prior studies in arid and semi-arid regions, as shown in Table 6. Consequently, across all locations examined, certain models display the capability to predict SPI with an accuracy level of no less than *R*^2^ 0.8. Comparing with the results obtained for other locations given in Table 6 in semi-arid regions with similar study sites (CPT, SB, UMT), it shows that for SPI3, Adnan et al. (2021) using RFVL–HGS model in Bangladesh obtained a correlation of 0.81, while Belayneh et al. (2016) obtained 0.88 using WNN in Ethiopia. Jalalkamali et al. (2015) obtained a correlation of 0.82 using MPL ANN, and Hosseini-Moghari et al. (2017) a correlation of 0.22 while using ICA-RSVR was obtained. Again, Kisi et al. (2019) reached a correlation of 0.87 using ANFIS-PSO. Subsequently, Gorgij et al. (2022) while using an LSTM with a range of parameters arrived at correlations ranging from 0.83 to 0.85. Comparatively, at this same timescale, the obtained results from our simulations for the different locations showed a better prediction except for SB (0.49–0.84). In the other locations, the correlation ranges from 0.77 to 0.95. Conversely, when SPEI 3 is in perspective, Khan et al. (2020) arrived at a correlation of 0.69–0.94), 0.76–0.79, and 0.60–0.71) with SVM, ANN, and KNN respectively over Rabi in Pakistan. So far, these results also do not diminish the performance of our models; rather, they show the robustness of our models. Furthermore, at SPI 6, other studies with different models obtained results as follows: Adnan et al. (2021) using RFVL–HGS in Bangladesh arrived at an *R*^2^ of 0.82, while Jalalkamali et al. (2015) obtained an *R*^2^ of 0.88 while using ANFIS in Iran. By applying ICA-RSVR in Iran as well, Hosseini-Moghari et al. (2017) had a result of 0.40 correlation. Also, Gorgij et al. (2022) and Kisi et al. (2019) obtained 0.88 to 0.90 and 0.87, with LSTM and ANFIS-PSO models, respectively. Aside SPI 6, models by Khan et al. (2020) and Mehr et al. (2023), which obtained similar results like our simulations SVM (0.95–0.97), ANN (0.87–0.91), KNN (0.65–0.86), and CNN-LSTM (0.68–0.75). In South Africa (Western Cape), Naik and Abiodun (2019) used the CORDEX to model SPI and SPEI to achieve a correlation of 0.24 and 0.38. Also, Mathivha et al. (2020) tested the accuracy range of models, GAM, EEMD-GAM, EEMD-ARIMA-GAM, and fORA at predicting SPEI which gave correlation of 0.48–0.95, 0.79–0.95, 0.94–0.99, and 0.92–0.99, respectively. For Deo et al. (2017), SPI was modelled using MARS, LSSVM, and M5Tree in Australia to obtain correlations of 0.98–0.99, 0.94–0.98, and 0.91–0.98, respectively. Furthermore, Habibia et al. (2018) while using the Asymmetric Power Autoregressive Conditional Heteroskedasticity (APARCH) time series model over the Chéliff–Zahrez basin in Algeria obtained a correlation ranging from 0.80 to 0.95 for an SPI-12 index. Subsequently, the results obtained from our simulations are also within the same range with the once already showcased, especially for Cape Town (0.57–0.95) and Umtata (0.61–0.87), while lesser accuracy was recorded at Spring Bok (0.22–0.75). The range of results in our simulations implies that the performance of some of our combination sets are at par with these other models Fig. 10.

Though, Naik and Abiodun (2019), Mathivha et al. (2020), and Deo et al. (2017) did not make a clear distinction of the temporal scales of their models, and our models compared well with them irrespective of the timescale. Subsequently, comparing with the results for an arid region in Iran obtained by Rafiei-Sardooi et al. (2018), which were *R*^2^ of 0.50 and 0.20 for Neuro-fuzzy and ARIMA respectively, it showed that both model performances were below the results we obtained for a similar location (Upington) which were 0.77–0.95 for SPI3 and 0.78–0.92 for SPI6. The range of results from our simulations implies that the performance of some of our combination sets are at par with what was obtained from these other models in both arid and semi-arid regions considered. Similar temporal variations observed in our simulation sets, between 3- and 6-month timescales, were also visible in other locations in which the differences are random. Such that a model may perform better in SPI3 and fail in SPI 6 and vice versa. Finally, the simulated results show that ANN drought simulations obtained for South Africa can compare effectively with results obtained for similar locations. Hence, the robustness and effectiveness of ANN in modelling drought at different temporal scales can be established.

### Comparison of measured and model SPI values

In this section, we compared the mean values of the measured and modelled results for the various locations at different timescales to ascertain the model with the most varying mean.

**Fig. 11:**
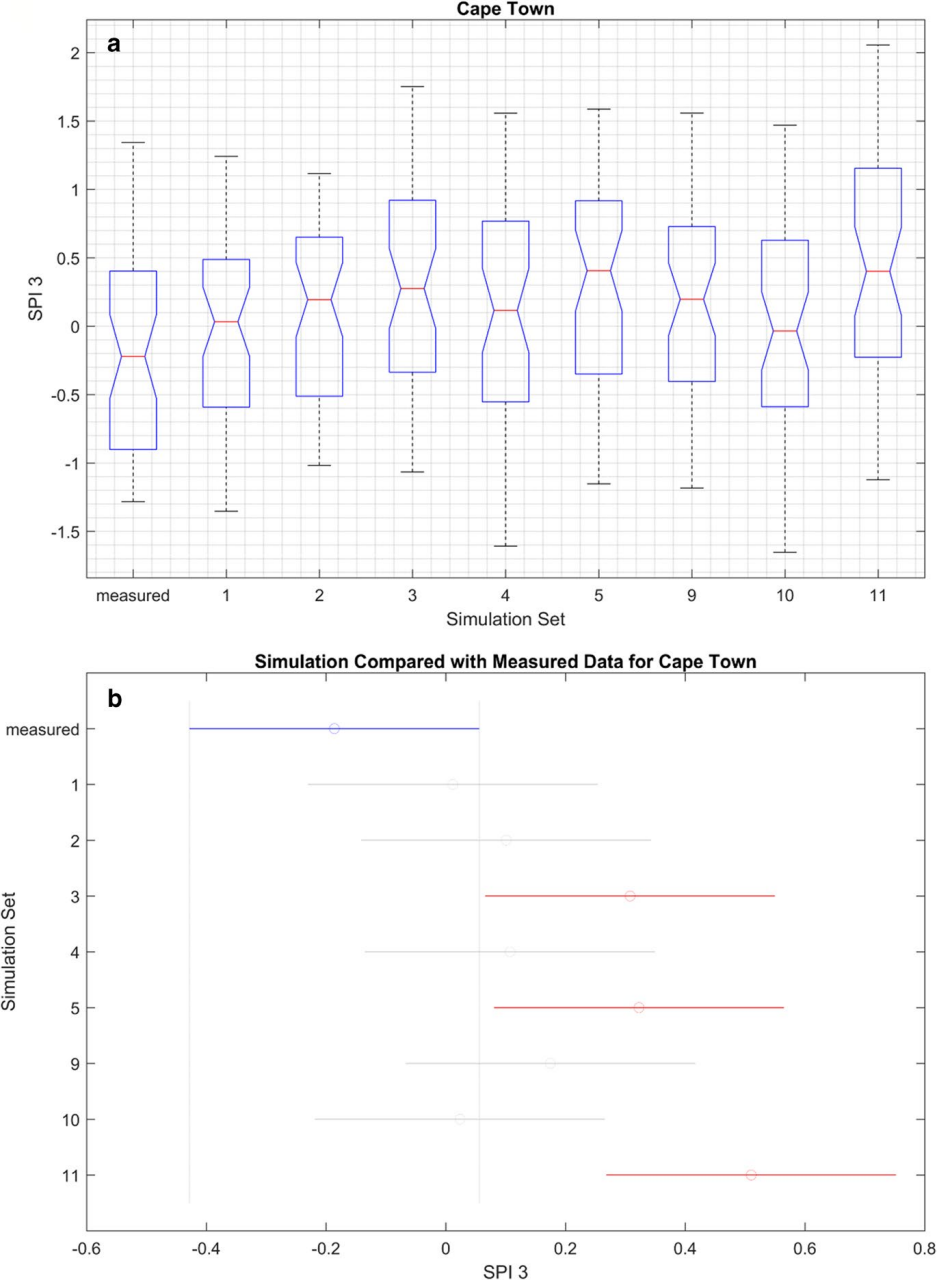
ANOVA plots representing the measured and modelled SPI 3 for Cape Town. The boxplots in **A** show the median for the different data sets while **B** is the mean, and the blue line represents the mean of the measured data, while the gray lines represent simulated data

**Fig. 12:**
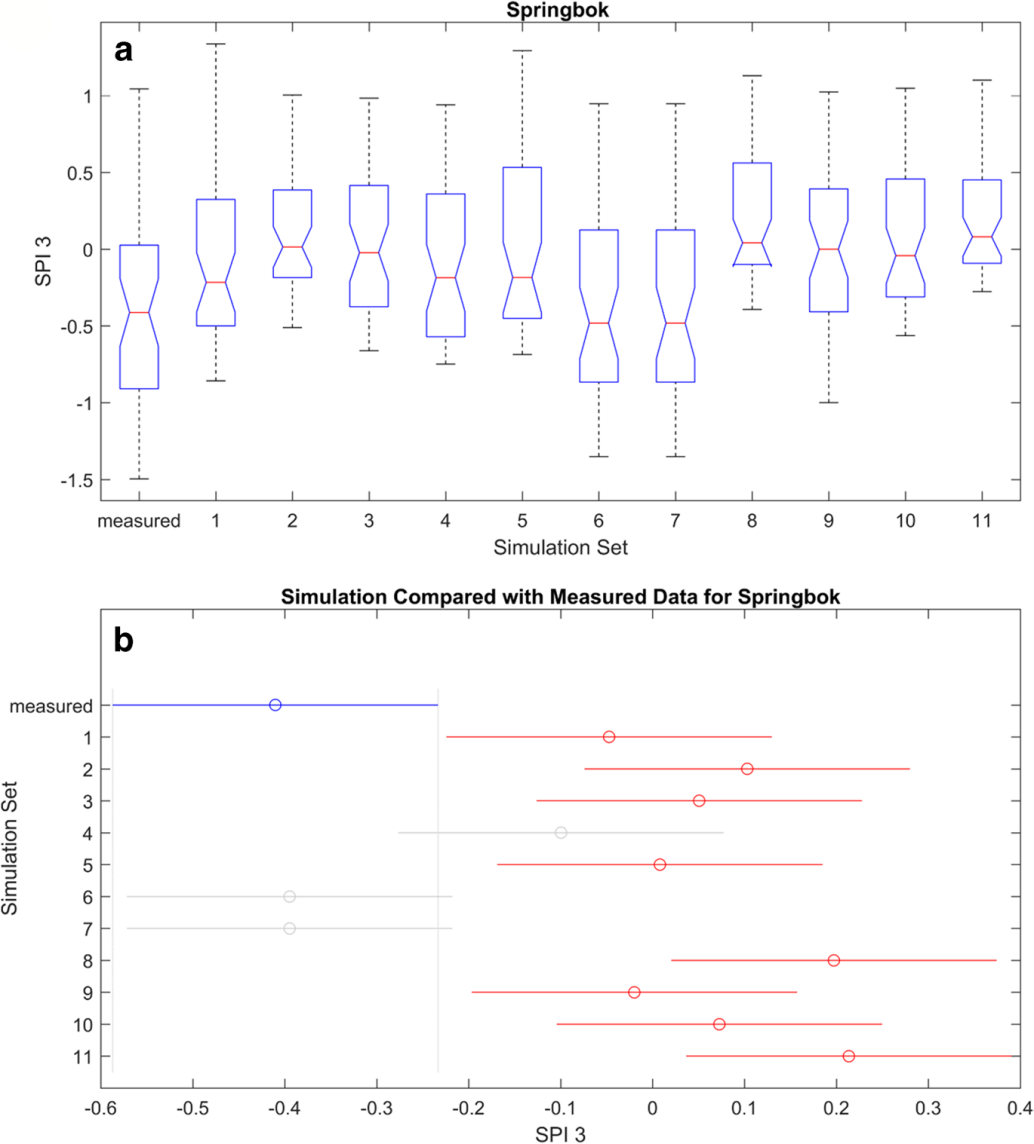
ANOVA plots representing the measured and modelled SPI 3 for Spring Bok. The boxplots in **A** show the median for the different data sets, while **B** is the mean, and the blue line represents the mean of the measured data, while the gray lines represent simulated data

**Fig. 13:**
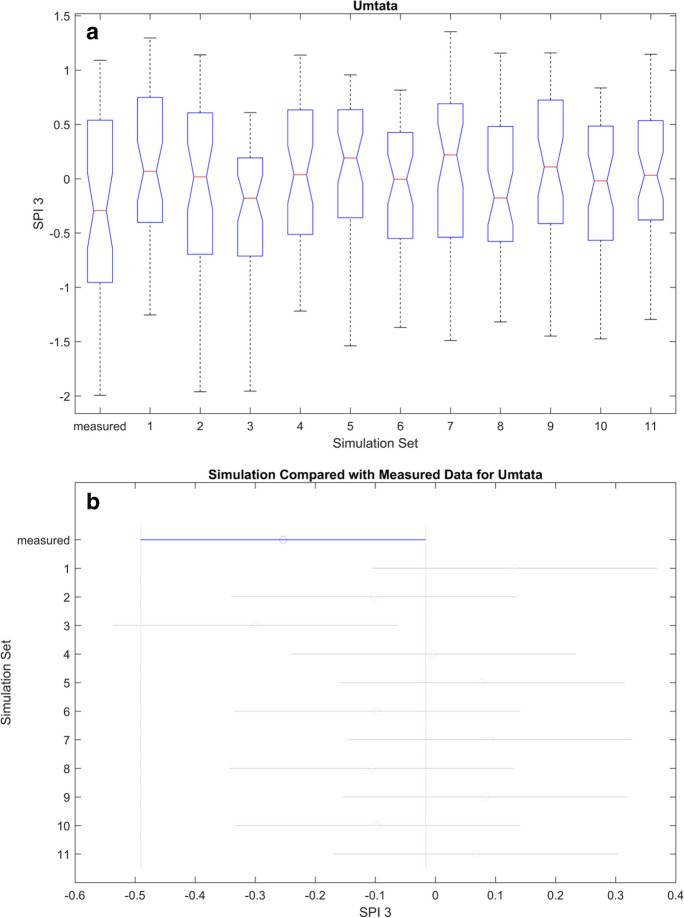
ANOVA plots representing the measured and modelled SPI 3 for Umtata. The boxplots in **A** show the median for the different data sets, while **B** is the mean, and the blue line represents the mean of the measured data, while the gray lines represent simulated data

**Fig. 14:**
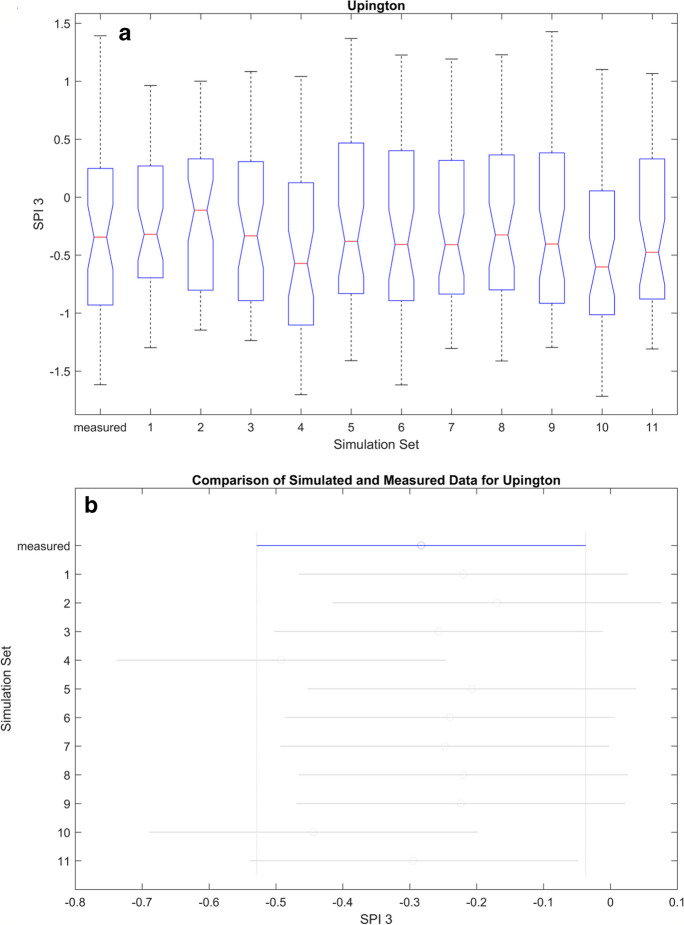
ANOVA plots representing the measured and modelled SPI 3 for Upington. The boxplots in **A** show the median for the different data sets, while **B** is the mean, and the blue line represents the mean of the measured data, while the gray lines represent simulated data. A comparison of the correlation and ANOVA results (Tables 9, 10, 11, and 12 in the Appendix and 8) show that high correlation may not correspond to similarity in the mean values. This does not invalidate the simulations as not being accurate; hence, both measurements are different

**Fig. 15:**
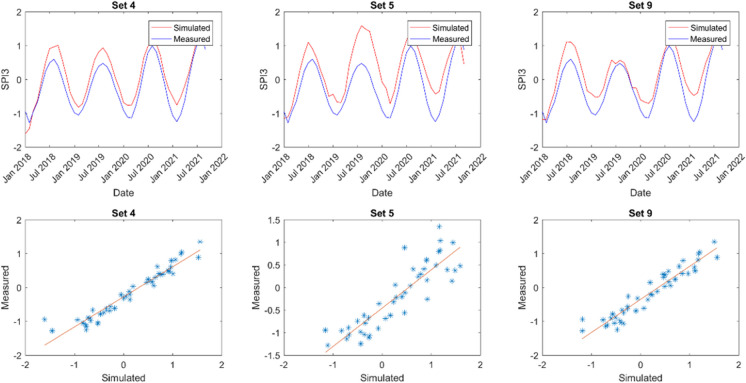
Simulations sets 4, 5, and 9 timeseries and scatter plots for CPT at SPI 3

**Fig. 16:**
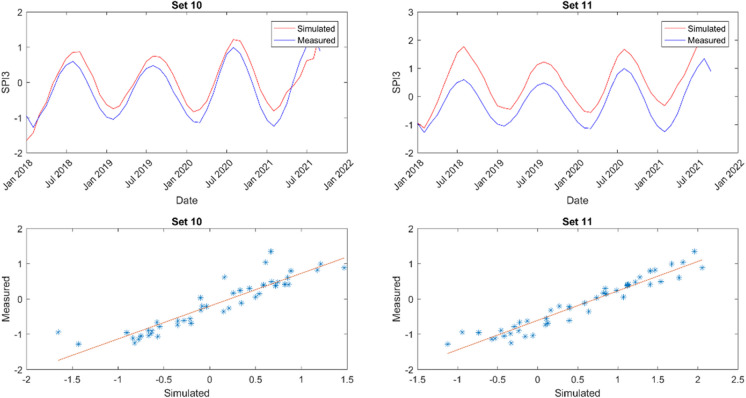
Simulation sets 10 and 11 timeseries and scatter plots for CPT at SPI 3

**Fig. 17:**
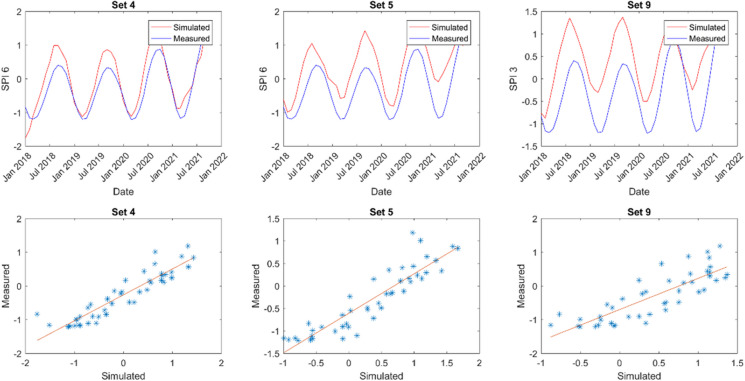
Simulation sets 4, 5, and 9 timeseries and scatter plots for CPT at SPI 6

**Fig. 18:**
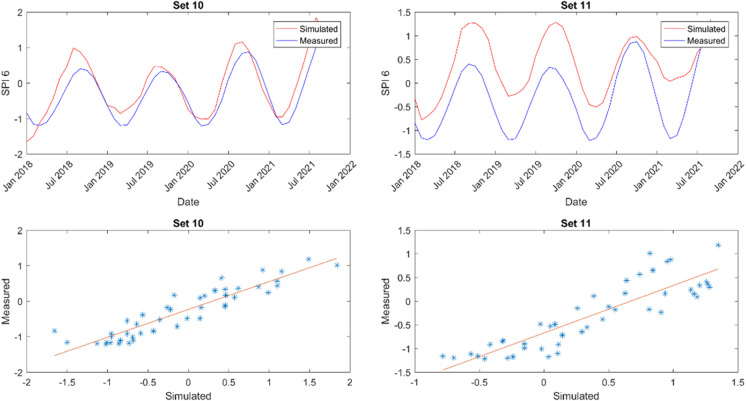
Simulation sets 10 and 11 timeseries and scatter plots for CPT at SPI 6

**Fig. 19:**
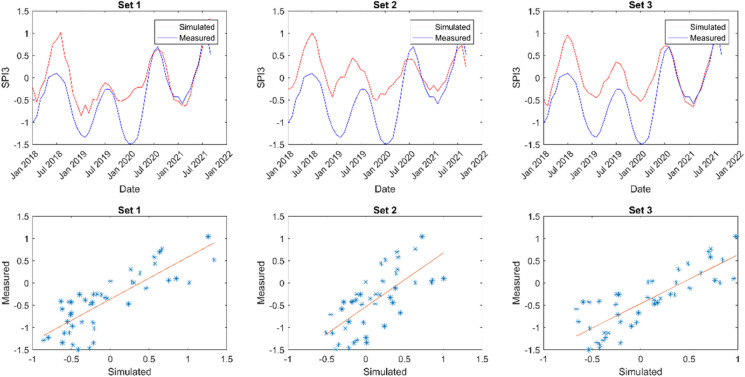
Simulation sets 1, 2, and 3 timeseries and scatter plots for Spring Bok at SPI 3

**Fig. 20:**
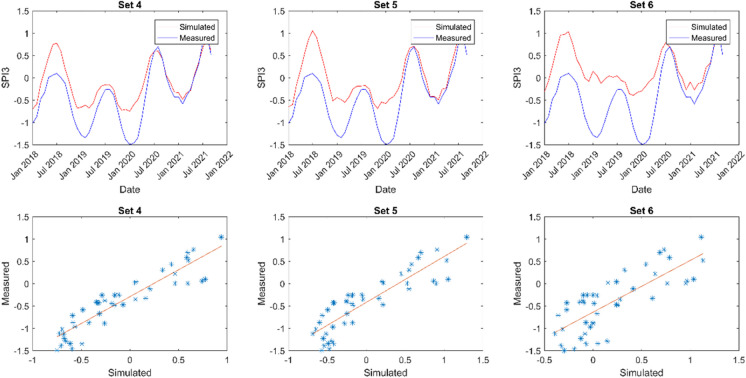
Simulation sets 4, 5, and 6 timeseries and scatter plots for Spring Bok at SPI 3

**Fig. 21:**
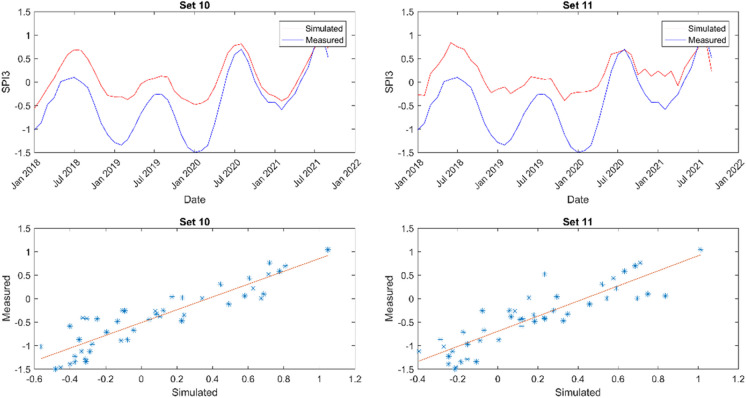
Simulation sets 10 and 11 timeseries and scatter plots for Spring Bok at SPI 3

**Fig. 22:**
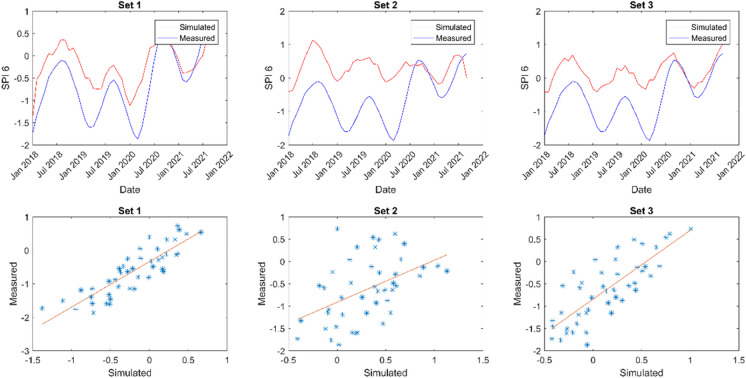
Simulation sets 1, 2, and 3 timeseries and scatter plots for Spring Bok at SPI 6

**Fig. 23:**
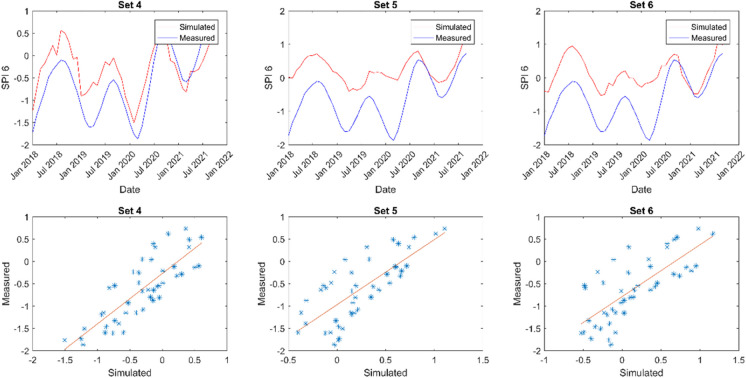
Simulation sets 4, 5, and 6 timeseries and scatter plots for Spring Bok at SPI 6

**Fig. 24:**
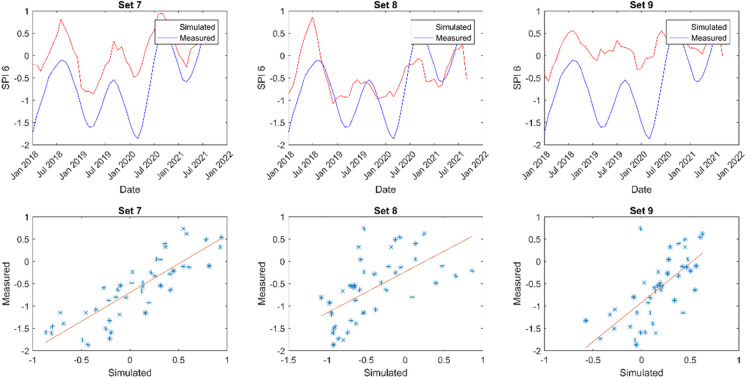
Simulation sets 7, 8, and 9 timeseries and scatter plots for Spring Bok at SPI 6

**Fig. 25:**
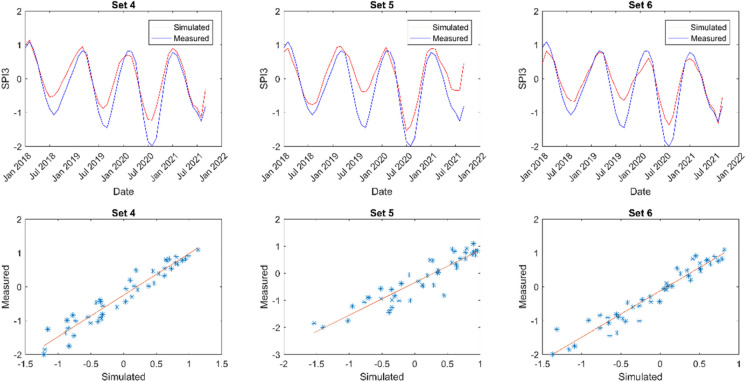
Simulation sets 4, 5 and 6 timeseries and scatter plots for Umtata at SPI 3

**Fig. 26:**
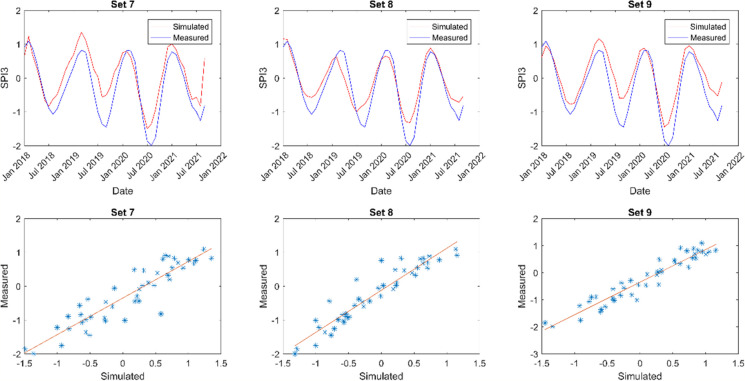
Simulation sets 7, 8, and 9 timeseries and scatter plots for Umtata at SPI 3

**Fig. 27:**
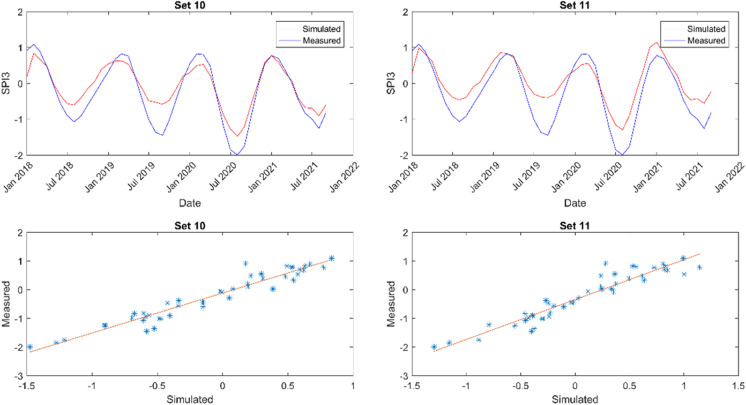
Simulation sets 10 and 11 timeseries and scatter plots for Umtata at SPI 3

**Fig. 28:**
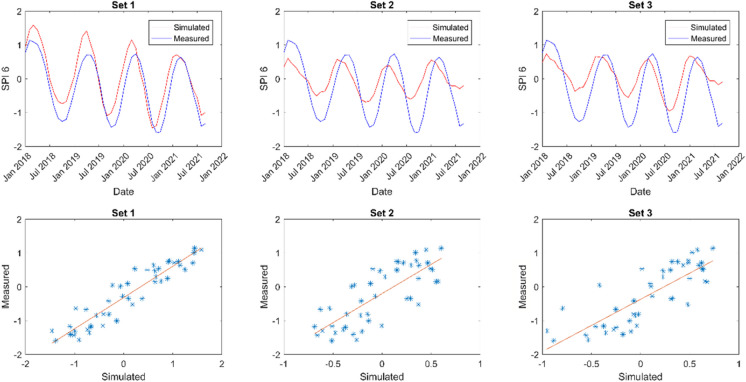
Simulation sets 1, 2, and 3 timeseries and scatter plots for Umtata at SPI 6

**Fig. 29:**
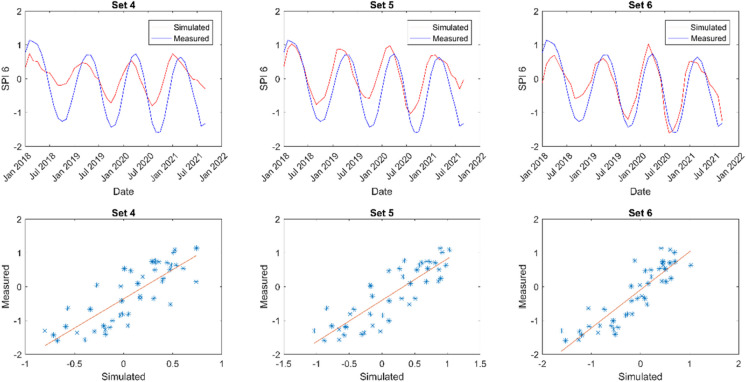
Simulation sets 4, 5, and 6 timeseries and scatter plots for Umtata at SPI 6

**Fig. 30:**
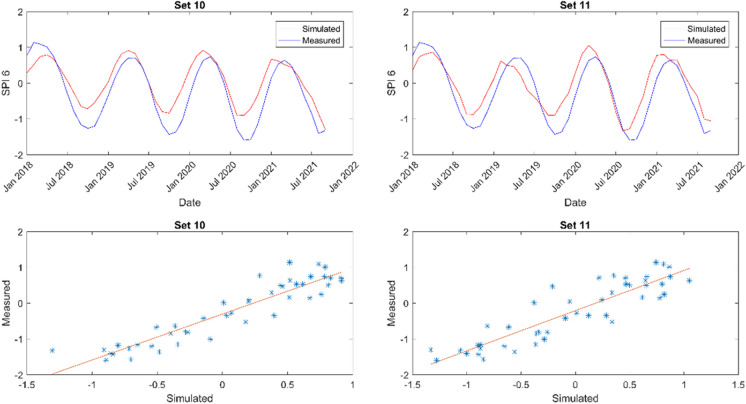
Simulation sets 10 and 11 timeseries and scatter plots for Umtata at SPI 6

**Fig. 31:**
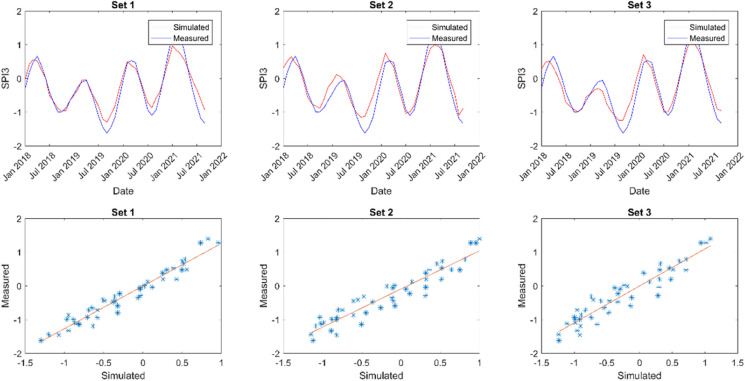
Simulation sets 1, 2, and 3 timeseries and scatter plots for Upington at SPI 3

**Fig. 32:**
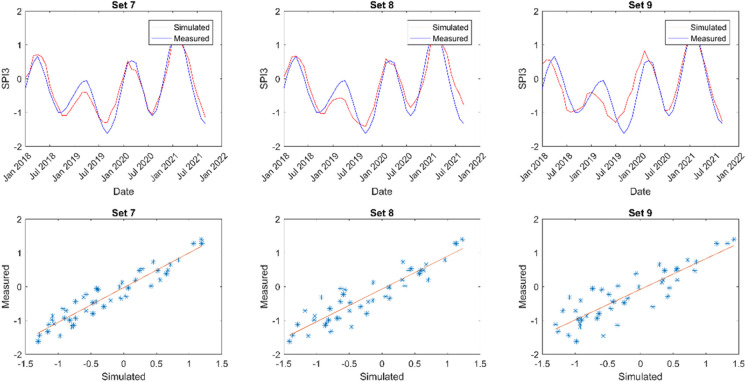
Simulation sets 7, 8, and 9 timeseries and scatter plots for Upington at SPI 3

**Fig. 33:**
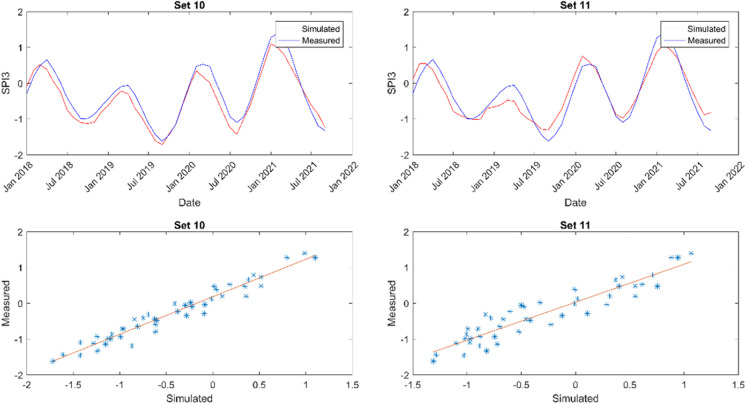
Simulation sets 10 and 11 timeseries and scatter plots for Upington at SPI 3

**Fig. 34:**
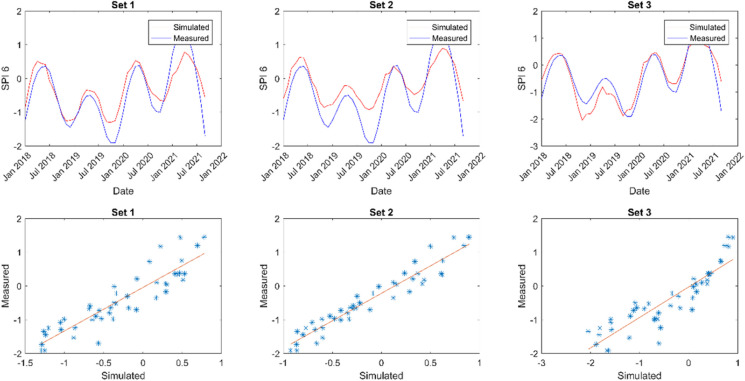
Simulation sets 1, 2, and 3 timeseries and scatter plots for Upington at SPI 6

**Fig. 35:**
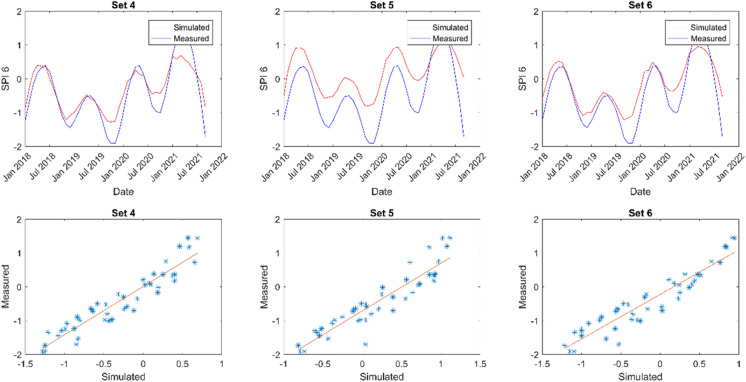
Simulation sets 4, 5, and 6 timeseries and scatter plots for Upington at SPI 6

**Fig. 36:**
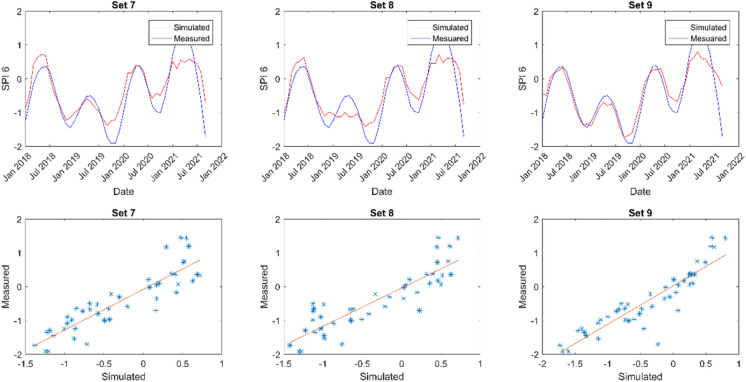
Simulation sets 7, 8, and 9 timeseries and scatter plots for Upington at SPI 6

**Fig. 37:**
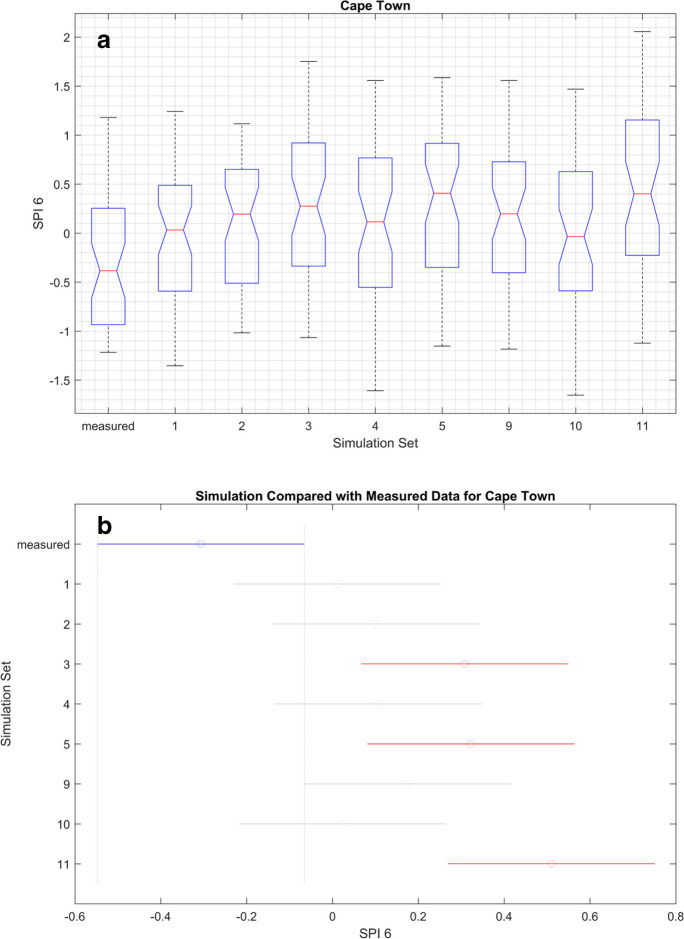
ANOVA plots representing the measured and modelled SPI 6 for Cape Town. The boxplots in **A** show the median for the different data sets, while **B** is the mean, and the blue line represents the mean of the measured data, while the gray lines represent simulated data

**Fig. 38:**
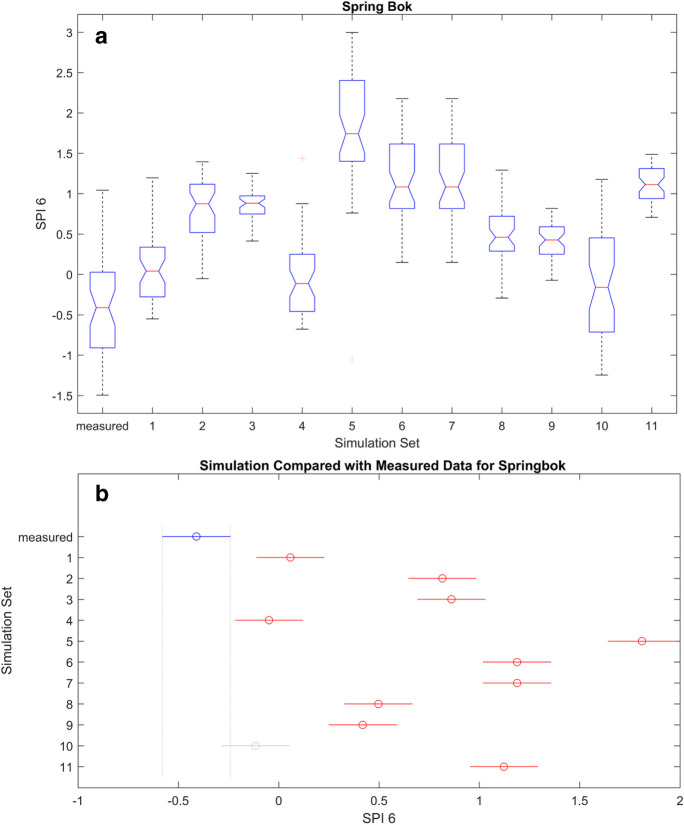
ANOVA plots representing the measured and modelled SPI 6 for Spting Bok. The boxplots in **A** show the median for the different data sets, while **B** is the mean, and the blue line represents the mean of the measured data, while the gray lines represent simulated data

**Fig. 39:**
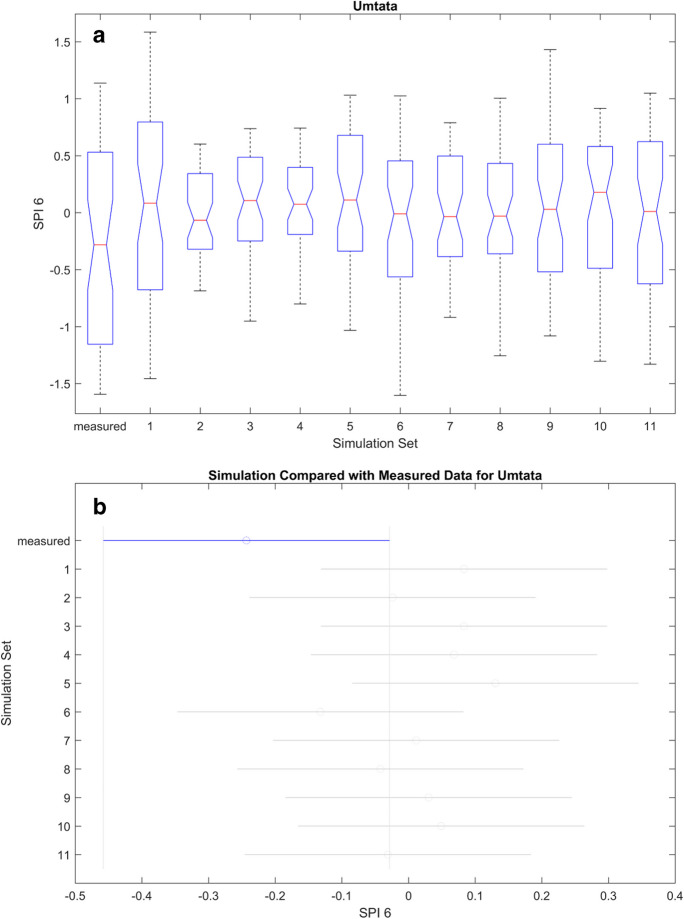
ANOVA plots representing the measured and modelled SPI 6 for Umtata. The boxplots in **A** show the median for the different data sets, while **B** is the mean, and the blue line represents the mean of the measured data, while the gray lines represent simulated data

Figures 11, 12, 13, and 14 (also refer to Figs. 37, 38, and 39 in the Appendix for the remaining ANOVA plots) illustrates ANOVA plots comparing the modelled and measured SPI. In the top row (box plots), the central mark represents the median (2nd quantile), while the edges denote the 25th and 75th percentiles. The whiskers extend to the most extreme data points, excluding outliers which are depicted by a + sign. The second row displays the comparison interval of the measured SPI (depicted by a blue bar) against simulations, where both differs it will be represented by a red bar.

**Fig. 40:**
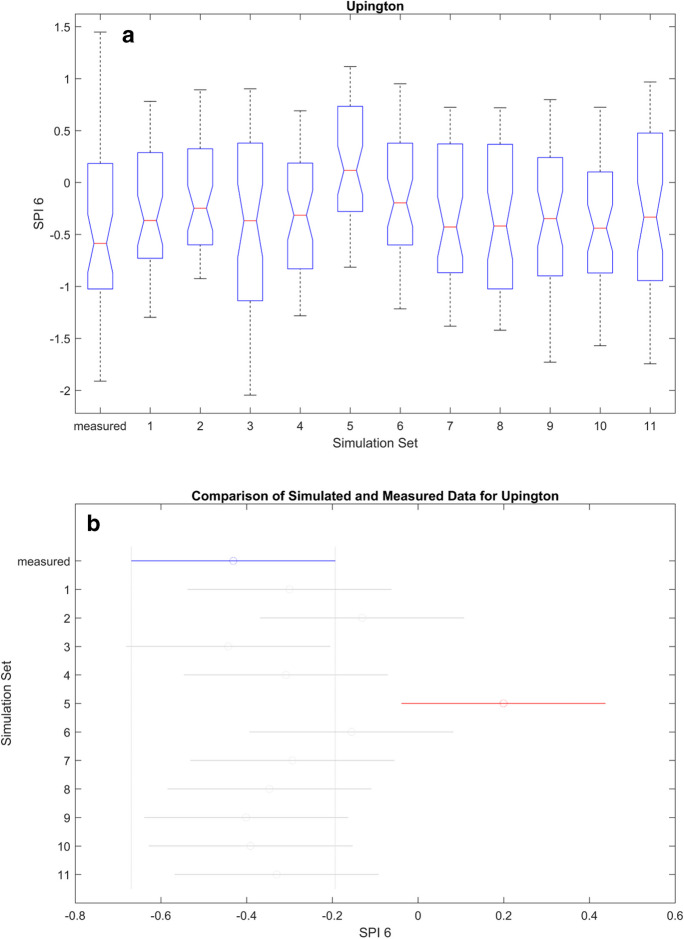
ANOVA plots representing the measured and modelled SPI 6 for Upington. The boxplots in **A** show the median for the different data sets, while **B** is the mean, and the blue line represents the mean of the measured data, while the gray lines represent simulated data

Table 7 shows that statistics of the ANOVA, while Table 8 summarizes the ANOVA results in both time scales as shown in Figs. 11, 12, 13, and 14 and Figs. 39 and 40 in the appendix. This result indicates how the mean values of the simulation set vary with the measured data and between each set. “Pass” and “fail” were used to denote the *p*-values, pass implies that all the simulation sets are within the same mean limit with the measured SPI and fail means at least one of the simulation sets has a mean that varied from the measured SPI. In that case, the null hypothesis is rejected whenever the *p*-value is “fail”.

**Table 7:** ANOVA statistic. *F*, *F*-statistic, *Prob* > *F*, *p*-value at 0.05 percentage confidence level

Location	SPI3		SPI6	
	*F*	Prob > F	*F*	Prob > F
Cape Town	3.46	Fail	4.47	fail
Spring Bok	8.35	Fail	15.17	fail
Umtata	1.86	Pass	1.25	pass
Upington	0.83	Pass	3.02	fail

**Table 8:** Summary of the ANOVA results; pass means that both predicated and measured results are similar while fail implies that both varied

Simulation sets	Cape Town		Spring Bok		Umtata		Upington	
	SPI 3	SPI6	SPI 3	SPI6	SPI 3	SPI6	SPI 3	SPI6
1	Pass	Pass	Fail	Fail	Pass	Pass	Pass	Pass
2	Pass	Pass	Fail	Fail	Pass	Pass	Pass	Pass
3	Fail	Fail	Fail	Fail	Pass	Pass	Pass	Pass
4	Pass	Pass	Pass	Pass	Pass	Pass	Pass	Pass
5	Fail	Fail	Fail	Fail	Pass	Pass	Pass	Fail
6	NR	NR	Fail	Fail	Pass	Pass	Pass	Pass
7	NR	NR	Pass	Fail	Pass	Pass	Pass	Pass
8	NR	NR	Pass	Pass	Pass	Pass	Fail	Pass
9	Pass	Fail	Fail	Fail	Pass	Pass	Pass	Pass
10	Pass	Pass	Fail	Pass	Pass	Pass	Pass	Pass
11	Fail	Fail	Fail	Fail	Pass	Pass	Pass	Pass

Table 7 shows that the mean values of all the simulations and the SPIs are similar at Umtata at both timescales and Upington at SPI 3. Subsequently, there are observed variations at Cape Town and Spring Bok at both timescales and Upington at SPI 6. Hence the summary in Table 8, where fail and pass are used to designate the simulations differs from the measured SPI. At CPT, it shows that simulations 3, 5, and 11 consistently varied at both timescales, while simulation 9 varied at SPI 6 only. Consequently, simulations 1, 2, 4, and 10 are consistent with the measured SPI at both timescales and simulation 9 at SPI 3 only. At SB, the mean values showed some variations at simulations 1, 2, 3, 5, 6, 9, and 11 at both timescales, and simulations 10 and 7 at SPI 3 and SPI 6 respectively, while they are similar at simulations 4 and 8 at both timescales, and at 7 for SPI3 and 10 for SPI 6. Furthermore, no variations were observed at Umtata, while only two variations were observed at Upington, simulation 8 for SPI 3 and simulation 5 for SPI 6.

Therefore, while a high correlation suggests a strong association between variables, it does not directly imply that their means will be similar. Hence, correlation measures the relationship or association, while means indicate the average values of the variables. This implies that although the means may differ, if the correlation is high, one variable might be predicted from the other with reasonable accuracy. This predictive power relies on the strength of the relationship despite their apparent mean estimates.

## Summary and conclusion

This study demonstrates that South Africa has been affected by drought of varying magnitudes over the past two decades, spanning from mild to extreme conditions. Regions like UM, SB, and UPT experienced mild droughts for over 40% of the time, while CPT encountered them for around 50% of the time. CPT also witnessed moderate drought for approximately 17.72% of the time at SPI3, followed by SB at 12.60%. In terms of severity, CPT faced moderate to extreme drought around 22% of the time, yet it received comparatively more rainfall, approximately 19%, compared to other locations. Overall, this investigation highlights that CPT and SB endured more frequent droughts than other areas studied, particularly those along the Atlantic coastlines.

The study utilized Artificial Neural Networks (ANN) to model drought in four South African locations, employing various meteorological and synoptic data combinations. The results revealed that ANN exhibited greater accuracy in predicting SPI3 compared to SPI6, when utilizing the same parameters within the same location. Moreover, models incorporating the evapotranspiration (et) parameter demonstrated better performance than those relying on evaporation (ev), suggesting et’s significance in drought prediction. Notably, a consistently high correlation was achieved from the “all” model across all locations and timescales. Similarly, models such as set3, set5, and set11 consistently modelled SPI more effectively compared to others.

Consequently, the study shows that ANN can be effectively used to model drought at different timescales; this is demonstrated by the accuracy and versatility of the results obtained. Therefore, researchers, policy makers, and practitioners can leverage on the accuracy of ANN to enhance their ability to simulate, manage, and put in place mitigation strategies to alleviate the impact of drought on both the economy and human water usage.

These results further emphasize the complexity and localization inherent in drought modelling, indicating that various parameters exhibit different performance levels across diverse locations. Consequently, the study emphasizes that a singular set of parameters cannot universally characterize drought modelling due to its multifaceted nature.

## Data Availability

Every data used in this research will be freely available on contacting any of the authors.

## Appendix

**Table 9:** Summary of outcomes obtained from the models for Cape Town

Cape Town	SPI 3			SPI 6		
Simulation	rmse	*R* ^2^	*p*-value	rmse	*R* ^2^	*p*-value
Set1	0.23	0.90	1.83e-23	0.156	0.95	5.91e-30
Set2	0.34	0.78	6.6e-16	0.474	0.57	4.2e-09
Set3	0.179	0.94	3.64e-28	0.37	0.73	8.64e-14
Set4	0.199	0.92	3.65e-26	0.279	0.85	4.66e-19
Set5	0.346	0.78	9.41e-16	0.295	0.83	5.14e-18
Set9	0.222	0.91	4.44e-24	0.398	0.69	2.01e-12
Set10	0.292	0.84	5.69e-19	0.272	0.85	1.59e-19
Set11	0.2	0.93	4.5e-26	0.339	0.77	2.01e-15

**Table 10:** Summary of results from the models for Spring Bok

Spring Bok	SPI 3			SPI 6		
Simulation	rmse	*R* ^2^	*p*-value	rmse	*R* ^2^	*p*-value
Set1	0.381	0.66	9.67e-12	0.36	0.75	1.6e-14
Set2	0.467	0.49	7.32e-08	0.636	0.22	0.00127
Set3	0.373	0.68	4.22e-12	0.437	0.63	7.86e-11
Set4	0.26	0.84	6.1e-19	0.407	0.67	3.47e-12
Set5	0.314	0.77	2.16e-15	0.476	0.56	3.25e-09
Set6	0.411	0.61	2.74e-10	0.479	0.58	3.99e-09
Set7	0.265	0.84	1.54e-18	0.38	0.72	1.84e-13
Set8	0.265	0.84	1.54e-18	0.569	0.37	8.34e-06
Set9	0.356	0.71	5.01e-13	0.52	0.48	1.53e-07
Set10	0.273	0.83	5.74e-18	0.43	0.64	3.76e-11
Set11	0.306	0.78	7.98e-16	0.40	0.68	2.55e-12

**Table 11:** Summary of results from the models for Umtata

Umtata	SPI 3			SPI 6		
Sets	rmse	*R* ^2^	*p*-value	rmse	*R* ^2^	*p*-value
Set1	0.197	0.95	2.26e-29	0.301	0.88	3.1e-21
Set2	0.304	0.88	2.86e-21	0.538	0.61	2.54e-10
Set3	0.367	0.82	1e-17	0.497	0.67	8e-12
Set4	0.229	0.93	1.42e-26	0.500	0.66	1.01e-11
Set5	0.343	0.85	5.42e-19	0.448	0.73	8.41e-14
Set6	0.238	0.93	8.25e-26	0.399	0.79	5.62e-16
Set7	0.361	0.83	5.35e-18	0.478	0.69	1.39e-12
Set8	0.291	0.89	4.69e-22	0.500	0.66	1.02e-11
Set9	0.257	0.91	2.22e-24	0.475	0.70	1.07e-12
Set10	0.234	0.93	3.81e-26	0.317	0.87	2.64e-20
Set11	0.276	0.90	5.03e-23	0.379	0.81	6.14e-17

**Table 12:** Summary of results from the models for Upington

Upington	SPI 3			SPI 6		
Combination	rmse	*R* ^2^	*p*-value	rmse	*R* ^2^	*p*-value
Set1	0.184	0.94	1.35e-28	0.388	0.81	3.09e-17
Set2	0.256	0.89	1.91e-22	0.256	0.92	5.6e-25
Set3	0.262	0.89	5.42e-22	0.402	0.80	1.43e-16
Set4	0.224	0.92	6.73e-25	0.301	0.89	5.71e-22
Set5	0.171	0.95	5.84e-30	0.343	0.85	1.63e-19
Set6	0.237	0.91	7.69e-24	0.305	0.88	9.85e-22
Set7	0.242	0.90	1.92e-23	0.413	0.79	4.75e-16
Set8	0.313	0.84	1.12e-18	0.421	0.78	1.13e-15
Set9	0.374	0.77	2.69e-15	0.384	0.82	1.98e-17
Set10	0.184	0.95	1.22e-28	0.29	0.90	1.05e-22
Set11	0.284	0.87	1.85e-20	0.324	0.87	1.32e-20
